# Novel Virulent Bacteriophages Infecting Mediterranean Isolates of the Plant Pest *Xylella fastidiosa* and *Xanthomonas albilineans*

**DOI:** 10.3390/v13050725

**Published:** 2021-04-21

**Authors:** Fernando Clavijo-Coppens, Nicolas Ginet, Sophie Cesbron, Martial Briand, Marie-Agnès Jacques, Mireille Ansaldi

**Affiliations:** 1 Laboratoire de Chimie Bactérienne, UMR7283, Centre National de la Recherche Scientifique, Aix-Marseille Université, 13009 Marseille, France; clavijo.d.f@gmail.com (F.C.-C.); nginet@imm.cnrs.fr (N.G.); 2 Institute Agro, INRAE, IRHS, SFR QUASAV, University of Angers, 49000 Angers, France; sophie.cesbron@inrae.fr (S.C.); martial.briand@inrae.fr (M.B.); marie-agnes.jacques@inrae.fr (M.-A.J.); 3 Bioline-Agrosciences, Equipe R&D–Innovation, 06560 Valbonne, France

**Keywords:** bacteriophage, *Xylella fastidiosa*, *Xanthomonas albilineans*, phage therapy, plant pathogens

## Abstract

*Xylella fastidiosa* (*Xf*) is a plant pathogen causing significant losses in agriculture worldwide. Originating from America, this bacterium caused recent epidemics in southern Europe and is thus considered an emerging pathogen. As the European regulations do not authorize antibiotic treatment in plants, alternative treatments are urgently needed to control the spread of the pathogen and eventually to cure infected crops. One such alternative is the use of phage therapy, developed more than 100 years ago to cure human dysentery and nowadays adapted to agriculture. The first step towards phage therapy is the isolation of the appropriate bacteriophages. With this goal, we searched for phages able to infect *Xf* strains that are endemic in the Mediterranean area. However, as *Xf* is truly a fastidious organism, we chose the phylogenetically closest and relatively fast-growing organism *X. albineans* as a surrogate host for the isolation step. Our results showed the isolation from various sources and preliminary characterization of several phages active on different *Xf* strains, namely, from the *fastidiosa* (*Xff*), *multiplex* (*Xfm*), and *pauca* (*Xfp*) subspecies, as well as on *X. albilineans*. We sequenced their genomes, described their genomic features, and provided a phylogeny analysis that allowed us to propose new taxonomic elements. Among the 14 genomes sequenced, we could identify two new phage species, belonging to two new genera of the *Caudoviricetes* order, namely, *Usmevirus* (*Podoviridae* family) and *Subavirus* (*Siphoviridae* family). Interestingly, no specific phages could be isolated from infected plant samples, whereas one was isolated from vector insects captured in a contaminated area, and several from surface and sewage waters from the Marseille area.

## 1. Introduction

*Xylella fastidiosa* (*Xf*) is a slow-growing Gram-negative plant-pathogenic bacterium emerging in Asia and Europe, and is therefore listed as a quarantine organism [1]. Long known in the Americas, this xylem-specialized bacterium is associated with numerous socio-economically important plant diseases. The impact of diseases due to *Xylella* include direct and indirect economic damages that were estimated to reach 150 million dollars per year in California when including measures to control vectors [2,3]. In the European olive oil production area, the projected impact of olive quick decline syndrome (OQDS) over 50 years ranges from two to five billion euros [4]. Nonetheless, such diseases have wider impacts; for instance, the impairment was estimated at 30% of the provision of ecosystem services provided by agroecological systems, including landscape value, cultural and natural heritage in the Puglia province of Italy [5]. *Xf* is transmitted by xylem sap-feeding insects and belongs to the *Xanthomonadaceae* (synonymous with the *Lysobacteraceae*) family. *Xf* genome has been the first plant pathogen genome sequenced, highlighting its importance as a plant pest [6]. Its genome of 2.6 Mb with a low GC content (52.7%) is smaller than that of other *Xanthomonadaceae*, even the ones of the phylogenetically closest relatives *Xanthomonas albilineans* (*Xa*) and *X. translucens* (*Xt*) whose genomes are composed of 3.7 and 4.7 Mb, respectively [7,8]. It is tempting to link the *Xf* reduced genome to its fastidious growth. Interestingly, a recent genome-scale metabolic network reconstruction analysis suggested that the *Xf* metabolic network is pretty much complete, although minimalist and not particularly robust [9]. Indeed, alternative reactions seem to be missing; the metabolism of the bacteria thus relies on poorly efficient metabolic pathways and lack of flexibility. Moreover, the study also found that the production of some virulence factors, such as exopolysaccharides (EPS), is at a high cost to the plant and is thus detrimental to fast growth [9].

*Xf* has been identified as the causative agent of Pierce’s disease, which has been causing extensive damage to vineyards in California for almost 150 years [10]. However, it was only at the end of the 1970s that this fastidious bacterium could be isolated on solid medium [11]. Since then, *Xf* has been associated with various forms of plant diseases on many different plant hosts [12]. Today, this bacterium belongs in the top 10 of the plant pathogens that are most studied worldwide [13]. *Xf* is endemic in the Americas and is persistently involved in large epidemics, causing significant damage in vineyards and citrus orchards. Listed as a quarantine pest in Europe, the three main *Xf* subspecies (*pauca* (*Xfp*), *multiplex* (*Xfm*) and subsp. *fastidiosa* (*Xff*)) were recently detected in Europe, in particular in the Mediterranean area, probably introduced through commercial exchanges and causing a variety of diseases such as the leaf scorch of olive trees in Italy [14]. Continuous monitoring using standardized real-time PCR protocols is now performed in southern Europe [15,16]. However, in the absence of any efficient and authorized chemical method to control *Xf*, a major challenge is to develop environmentally friendly biotechnologies to control this plant disease. The current strategy implemented by the European Commission to prevent *Xf* spread within the EU consists of eradication and containment measures involving intensive surveillance, vector control treatments, and differential removal of infected plants and specified hosts in foci and buffer zones [17]. Additional measures to these economically and socially devastating ones have been proposed, such as the selection of naturally resistant host plants, the thermal treatment of young plants, or the use of a combination of zinc, copper, manganese and citric acid [18,19,20]. Biocontrol strategies have also been proposed using avirulent *Xf* strains or other avirulent plant colonizers [21,22]. However, none of these later strategies has been recognized as fully efficient or totally safe by the European authorities.

Bacteriophages, the viruses infecting bacteria, have been used to treat infectious diseases since their discovery at the beginning of the twentieth century [23]. Their use in therapy has pros and cons, but the clear advantages are that they can infect antibiotic-resistant pathogens and can be adapted to any bacterial species as long as strictly virulent phages can be isolated, which can be difficult with some poorly growing species. Recently, the agricultural sciences community put a great deal of emphasis on phage therapy to develop sustainable strategies of biocontrol for plants and animals [24,25,26]. Currently, a number of phage products are being developed or are already commercially available for a variety of plant pathogens such as various *Xanthomonas* strains or *Ralstonia solanacearum* [27]. As an example, the OmniLytics™ company commercialized several phage products under the AgriPhage™ name to control tomato and pepper bacterial spot and speck caused by *Xanthomonas euvesicatoria* pv. *euvesicatoria* and *Pseudomonas syringae* pv. *tomato,* as well as other plant diseases. In this context, a group from Texas A&M University proposed the use of bacteriophages as a treatment for Pierce’s disease [28,29,30]. Following the same line of investigation, we attempted to isolate *Xf*-specific phages and to focus on the three *Xf* subspecies present in the Mediterranean basin, since the strains currently spreading in Europe differ from the original Pierce’s disease causative strain *Xff* [14,16]. In order to develop phage cocktails active against specific strains of interest, i.e., *Xf* strains endemic in Europe, one should first constitute a collection of dedicated and specific phages, determine their genome sequences and test them in vitro as well as in vivo for their lytic activity. In this work, we started to build up a phage library that could eventually be used as biocontrol products.

## 2. Materials and Methods

### 2.1. Bacterial Strains and Media

The bacterial strains used in this study are listed in Table 1 and were provided by the French Collection of Plant-associated Bacteria (CIRM-CFBP, https://www6.inrae.fr/cirm_eng/CFBP-Plant-Associated-Bacteria, accessed on 10 March 2021). *Xf* strains were cul-tured at 28 °C on modified periwinkle wilt modified medium (PWG-M agar without phenol red) [31] for up to seven days. Then, the cultures were transferred to a new plate containing PWG-M agar or B-CYE medium [32] and incubated for up to seven days at 28 °C. When required, for liquid cultures, *Xf* strains were incubated at 28 °C under 160 rpm agitation in PD2 broth [31]. Xanthomonas strains were cultured on YPG agar (yeast extract, 7 g l-1; peptone, 7 g l-1; glucose, 7 g l-1; agar 15 g l-1 (pH 7.0 to 7.2)) [33] for up to 24 h at 28 °C, except for *X. arboricola* (*Xaj*) and *X. albilineans* (*Xa*) cultured for two to three days at 28 °C. For liquid cultures, *Xanthomonas* strains were cultured in YPG broth medium (YPG without agar). The YPG soft agar medium (YPG broth with agar 7.5 g l-1) was used for *X. albilineans* overlays at 28 °C for up to three days. For all strains, suspensions made from fresh cultures were stored at −80 °C in YP glycerol medium (yeast extract 5 g l-1; peptone 5 g l-1; glycerol 30% *v*/*v*), except for the *X. albilineans* strain, which was stored directly in sterile distilled water.

### 2.2. Environmental Samples for Phage Isolation

Several Xylella-associated and Xylella-non associated environments were assayed for the presence of bacteriophages. For Xf-associated environments, plant samples were collected in a Corsican public space (GPS coordinates 41.912444, 8.641105), and sap-feeding insect samples (of the species Philaenus spumarius) were collected nearby (GPS coordinates 42.272662, 9.492568). Both positions are referenced as Xylella-infected locations in France [34]. Samples were sterilized on-site as follows: plant and insect extracts were macerated in 5 mL of phage buffer (100 mM Tris-HCl (pH 7.6); 100 mM NaCl; 10 mM MgCl_2_; and 10 mM MgSO_4_) per gram of material. To remove large particles, the mixtures were filtered through a 320 mm Whatman^®^ filter paper, Grade 113V. Supernatants were filtered through 0.45 μm then through 0.22 μm pore-size nylon Acrodisc^®^ syringe filters, and then stored at 4 °C in the presence of a few drops of chloroform.

For *Xylella*-non-associated environments, one liter of sewage influent was collected from the wastewater treatment plant of Marseille Provence Metropole, and one liter of post-rain runoff waters was collected from Marseille Vieux-Port, France (GPS coordinates 43.2941456, 5.373712). Large particles were removed as above. Bacteriophage particles were then pelleted by high-speed centrifugation at 90,000× *g* for one hour at 4 °C. Pellets were suspended in 10 mL phage buffer and stored at 4 °C. Since we noticed that any chloroform addition to the samples led to a reduced number of plaques, chloroform was not added to the water samples. Runoff water sampling did not require specific permissions, whereas, for raw sewage samples, approval from Marseille Provence Metropole was obtained before sampling.

### 2.3. Phage Enrichment

**Figure 1:**
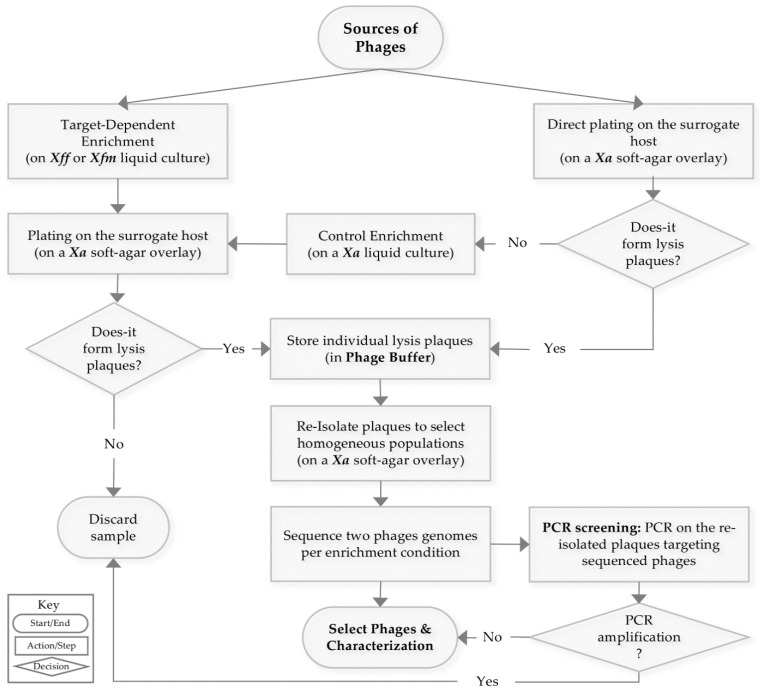
Workflow for phage isolation. This pipeline represents the principal steps and methods used to isolate bacteriophages active on *X. fastidiosa* (*Xf*), using *X. albilineans* (*Xa*) as a surrogate host to propagate phages. The workflow begins by enriching extracts of plants, insects, sewage waters, and runoff waters on *Xf* subspecies *fastidiosa* (*Xff*) and Scheme 56. were re-isolated at least three times to obtain uniform lysis plaques, and 14 plaques were treated to obtain individual phages for sequencing. Phage DNA was extracted and sequenced using our in-house Illumina platform. The DNA sequences were processed, and specific primers targeting the sequenced phages were designed. These primers were used to directly PCR-amplify the DNA present in the 56 purified plaques. Sequenced phages and new candidates from the PCR screening were considered for further phage characterization.

Enrichments from the plant, insect, sewage and runoff water samples were conducted in two ways: on *Xylella fastidiosa* (*Xf*) cultures, to select directly for *Xf*-specific phages, and on *X. albilineans* (*Xa*) cultures to maximize our chances of isolating *Xanthomonaceae*-infecting phages. *Xf* strains were grown at 28 °C on PWG-M agar medium [31] for up to 15 days and transferred to 2 mL PD2 broth medium, to which 200 μL of pre-treated samples were added. The culture enrichments were incubated for 10 days at 28 °C without agitation. Plant and insect extracts were also enriched on the surrogate host *Xa* as follows: *Xa* strain CFBP 2523 was cultured overnight in 5 mL LPG broth at 28 °C under 160 rpm agitation. Two ml of *Xa* liquid culture in YPG broth was calibrated at an OD_600_ of 0.2 and incubated with 200 μL of pre-treated samples for two days at ~25 °C without agitation. *Xa* and *Xf* cells were then removed by centrifugation at 4000× *g* for 15 min, and the supernatants were filtered through a 0.22 μm pore-size Acrodisc^®^ filter. Enrichment products were stored in sterile conditions at 4 °C. To be noted, filtered wastewater and runoff samples formed lysis plaques on a *Xa* overlay without any enrichment step. Phage enrichment was confirmed, and the phages were re-isolated using plaque assay on *Xa* overlays. A descriptive workflow of our strategy is illustrated in Figure 1.

### 2.4. Phage Isolation, Propagation and Purification

Phage activity was tested using spot assay on double agar overlay plates, using the surrogate host *X. albilineans.* Briefly, *Xa* was cultured for 8 to 12 h on YPG broth at 28 °C under 160 rpm agitation. Then, 200 μL of bacterial culture were mixed with 2.8 mL of YPG soft agar at fusion temperatures close to 50 °C. The mixture was vortexed and plated on YPG agar plates. Lytic activity was revealed by exposing *Xa* soft agar overlays to serial 10-fold dilutions of the enrichment products and incubated for two to three days at 28 °C. Phage-forming lysis plaques were selected and plugged off from the agar surface bottom using a 1000 µL tip, and then suspended in 500 µL of phage buffer. Re-isolation was performed on a *Xa* overlay at least three times, repeating plugging and suspension steps to select single plaques with a regular shape. At the third re-isolation step, phage particles were propagated by adding 100 μL of phage suspension to a 6 mL *Xa* liquid culture in LPG broth calibrated at an OD_600_ of 0.2. After two days of incubation at 28 °C under 160 rpm shaking, cells were removed by centrifugation at 5000× *g* for 15 min and supernatants were filtered through a 0.22 μm pore size Acrodisc^®^ syringe filter. For phage purification, phage particles were pelleted at least three times by centrifugation at 20,800× *g* for 1 h at 4 °C, then suspended in phage buffer. Final suspensions were filtered through a 0.22 μm filter and stored in sterile conditions at 4 °C.

### 2.5. Negative Staining and Transmission Electron Microscopy (TEM)

Phage preparations (2 mL) were pelleted by a mere centrifugation step at 20,800× *g*, 4 °C for one hour. Pelleted phages were washed and pelleted again three times with 2 mL of TEM-buffer (0.22 µm filtered 0.1 M NH4-acetate (pH 7)) and concentrated in a final volume of 50 μL TEM-buffer. Then, five μL drops of diluted phage suspensions (~2 × 10^8^ PFU·mL^−1^) were spotted on glow discharged carbon-coated grids (EMS) and let stand for 3 min. The grids were then washed with two drops of 2% aqueous uranyl acetate and stained with a third drop for 2 min. Grids were dried on filter paper, and the samples were analyzed using a Tecnai 200 KV electron microscope (FEI). Digital acquisitions were made with a numeric camera (Eagle, FEI). Electron micrograph images were analyzed using the ImageJ software [35].

### 2.6. Phage Genome Sequencing and Genetic Analysis

Total phage DNA was extracted using the classical phenol/chloroform extraction method, then sequenced using the MiSeq Illumina^®^ technology. The phage DNA li-braries were prepared using NexteraTM enzymatic fragmentation technology, a tech-nology suitable for double-stranded DNA (dsDNA). Paired-end reads were assembled with SOAPdenovo, version 2.04 [36] and VELVET, version 1.2.10 [36,37]. Whole se-quences were compared to the NCBI BLASTn database. Protein coding sequences, ge-nomic size and GC content were predicted by the RAST^®^ tool [38]. Predicted CDS were compared with the NCBI BLASTp database, NCBI conserved domain database and HHpred (PDB_mmCIF73_22_May database).

Genomic comparisons were performed with shared *k*-mer analysis using the KI-S protocol and the similarity matrix was represented using the KI-S circle packing tool [39] available at the CIRM-CFBP Galaxy platform (https://iris.angers.inra.fr/galaxypub-cfbp/, accessed on 28 March 2019).

Phylogenetic analyses were generated by the VICTOR online tool [40] using the complete genomic sequences of the *Caudovirales* bacteriophages infecting Xanthomonadales present in Genbank (Supplemental Table S1). All pairwise comparisons of the amino acid sequences were conducted using the Genome-BLAST Distance Phylogeny (GBDP) and trees were inferred under settings recommended for prokaryotic viruses [40]. Taxon boundaries at the species, genus and family level were estimated with the OPTSIL program [41].

### 2.7. Phage Host Range

Host ranges of isolated phages were determined using the phage sensitivity spot test as follows: *X. fastidiosa* strains (Table 1) were cultured at 28 °C on PWG-M agar plates for up to seven days. Then, the cultures were suspended in sterile distilled water and OD_600_ was calibrated at 0.1 for *Xfp* and *Xfm* and 0.01 for *Xff* strain. Drops of 20 μL of bacterial suspensions were deposited on the surface of the B-CYE medium [31] and allowed to dry. Then, 10 μL of purified phage preparations (10^10^ PFU·mL^−1^) were deposited at the margin of the upper part of the bacterial drop. As a control for each strain, one bacterial drop did not receive any treatment, and one bacterial drop was assayed with 10 μL of phage buffer. Spots were dried at room temperature and the plates cultured for up to 10 days at 28 °C. All experiments were performed in triplicate.

### 2.8. Phage Nomenclature and Nucleotide Sequence Accession Numbers

Phages isolated in this study were named according to the classification guide proposed by Adriaenssens and Brister [42]. Note that the names of the newly isolated phages originate from neighborhood names in the city of Bogota, Colombia. The assembled genome sequences, as well as raw reads, were deposited at the European Nucleotide Archive (ENA) and the National Center for Biotechnology Information (NCBI) under the accession numbers listed in Table 2.

## 3. Results

### 3.1. Strategy to Isolate Xf-Specific Phages

To bypass the fastidious in vitro growth of *Xf*, we developed a strategy using *Xa* as a surrogate host to isolate phages. Using surrogate hosts for bacteriophage isolation and propagation was proposed by several studies to isolate phages against pathogenic bacteria such as *S. aureus* [43,44], *M. tuberculosis* [45], and *S. enterica* [46], as well as for the plant pest *X. fastidiosa* [28]. To select bacteriophages with a lytic activity on *Xf*, samples from the plant, insect, sewage and runoff waters were enriched on liquid cultures of *Xff* and *Xfm* strains. All samples were also exposed directly to *Xa* soft-agar overlays, either directly or previously enriched on *Xa* cultures. None of the plant extracts, either plated directly on to *Xa* lawns or after enrichments on *Xff*, *Xfm* or *Xa*, were able to form lysis plaques on the surrogate host overlays. In the same way, insect extracts plated directly on a *Xa* overlay were not capable of forming lysis plaques. In contrast, insect extracts enriched either on *Xff*, *Xfm* or *Xa* liquid cultures led to the isolation of viral particles able to form plaques on *Xa* overlays. Regarding the *Xylella*-non-associated environmental samples, sewage water led to the formation of numerous plaques on *Xa* overlays without any enrichment step, indicating a wide variety of *Xa*-infecting phages in these samples. The same situation was observed for the sewage samples previously enriched on *Xff* and *Xfm*. We re-isolated six plaques of each *X. fastidiosa* enrichments (6 + 6) and 12 for *Xa* control enrichment (Table 3). Runoff water samples enriched on *Xff* and *Xfm* did not form any plaque on *Xa* overlays, while raw extracts were able to form plaques without a prior enrichment step. Eight phage plaques were re-isolated in this way. Two phage genomes obtained in each experiment were sequenced immediately, leading to an initial set of 14 sequenced phage genomes.

Based on their DNA sequences, we divided this initial set of 14 genomes into four groups based on DNA homology, using a cutoff of more than 90% of nucleotide identity and a coverage of more than 80% to allow a highly diverse phage library build-up. These groups are represented by phages FC03-Usme, FC12-Bacata, FC23-Cota and FC44-Bosa. This allowed us to design four primer pairs specific to each group, in order to confirm the affiliation of the other 10 phages as sequenced to their proper group. Furthermore, with these four sets of primers we also tested all remaining plaques isolated in this study (56 in total). This strategy aimed to rapidly eliminate phages that are already sequenced and identify new phages to sequence.

Our PCR screening yielded the following results (Table 3). Among the 24 phages isolated from insect extracts, 23 were FC03-Usme-like phages, while one belonged to the FC44-Bosa group. Among the 12 phages isolated using the sewage water samples, we obtained without enrichment four FC03-Usme-like phages, four FC44-Bosa-like phages, and two FC12-Bacata-like phages. Interestingly, among these 12 newly isolated phages, FC41-Suba and FC42 were not amplified by any of the specific primer pairs designed for the four initial groups, suggesting new genome sequences; FC41-Suba DNA was further extracted and sequenced. Similar sequence analysis led us to conclude that FC41-Suba represented a new group of phages. All six re-isolated phages obtained from the sewage water extracts after enrichment on *Xff* were identified as FC12-Bacata-like phages. However, two amplification profiles also revealed a positive amplification with FC44-Bosa primers, suggesting that both phages were present on the re-isolated plaques, probably by cross-contamination. Interestingly, these results suggest that both FC12-Bacata-like and FC44-Bosa-like phages were able to multiply on *Xylella* strains during the enrichment step. Among the six phages isolated on sewage water samples enriched on *Xfm*, we identified by PCR screening three FC23-Cota-like, one FC03-Usme-like and one FC44-Bosa-like phages. Interestingly, more diversity was obtained on sewage water samples enriched on *Xfm*, and none of the primer pairs designed did amplify on phage FC24-Teja. Unfortunately, and despite extensive efforts, DNA from this phage was impossible to extract, suggesting that its genome is composed either of highly modified DNA that cannot be extracted in the same way as the other phage DNAs, or is composed of another type of nucleic acid molecule. Of note, we have not yet tried to extract RNA from this phage preparation. We were thus not able to obtain the sequence of this particular phage genome. Finally, among the eight phages isolated from runoff water samples without enrichment, we found four FC03-Usme-like phages and four phage isolates whose DNA could not be amplified with any primer pair, indicating either a different type of nucleic acid, or that they constitute one to four new groups of phages that were designated FC32-Sibate_a, FC32-Sibate_b, FC34-Cajica_a and FC34-Cajica_b (a and b indicate two different plaque morphologies on *Xa* overlays).

At this stage, our PCR screening method enabled us to isolate at least five different PCR groups of dsDNA bacteriophages represented by phages FC03-Usme, FC12-Bacata, FC23-Cota, FC41-Suba and FC44-Bosa. Three other bacteriophage isolates, represented by FC24-Teja, FC32-Sibate and FC34-Cajica, were potential new phages; their sequences remain to be obtained to determine their nature.

### 3.2. Genomic Diversity of the Phages Isolated

According to our method, 10/14 sequenced phages and 32/56 phages screened by PCR belong to the FC03-Usme-like phage group, the most abundant group isolated in this study. By comparing the complete DNA sequences (BLASTn analysis), we observed that the FC03-Usme genome shares 100% identity with 99% coverage with the FC08-Olaya, FC15-Bolivar, FC17-Usaquen, FC25-Alcala, FC47-Fontebon and FC57-Sumapaz genomes, indicating that these phages were different isolates of the same phage species using the species demarcation criteria defined by the International Committee on Taxonomy of Viruses (ICTV). The FC28-Sopo, FC30-Tabio and FC39-Tenjo genomes were similar to FC03-Usme, with 91–92% identity and a coverage of 89%. FC23-Cota, FC41-Suba and FC44-Bosa were phage singletons, and their DNA sequence could not be associated at the DNA level with any other phage DNA sequenced in this study.

**Figure 2:**
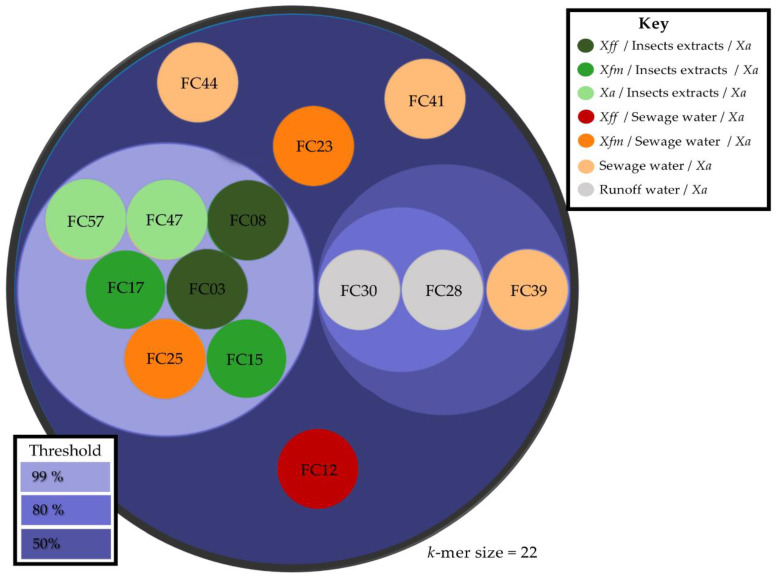
Circle packing visualization of shared *k*-mer analysis. Pairwise similarities between whole phage genomes sequenced in this study clustered according to their shared 22-mers. Circles representing each phage genome were colored according to the isolation conditions of each phage. Inner and outer circles are based on shared *k*-mer thresholds of 99% (light purple), 80% (medium purple) and 50% (dark purple), respectively. The FC03 group contains the isolated phages FC03-Usme, FC08-Olaya, FC15-Bolivar, FC17-Usaquen, FC25-Alcala, FC28-Sopo, FC30-Tabio, FC47-Fontebon and FC57-Sumapaz and forms two different subgroups. FC23-Cota, FC41-Suba and FC44-Bosa are neither associated with any of the previous groups, nor with each other. The matrix of shared *k*-mers is presented in Table S2.

In order to obtain more information on the diversity of the 14 phage genomes sequenced thus far, we performed a comparative study based on the analysis of shared oligonucleotides signatures (*k*-mers) using the KI-S tool [39]. Complete DNA sequences were compared, and the percentage of shared 22-mers was determined, allowing us to group the phage genomes into connected component clusters using shared *k*-mer thresholds of 99%, 80% and 50% (Figure 2 and Table S2). The evaluation of the phage variant synonymy, using the discriminatory selection of the 22-mers, suggested that the 10 FC03-Usme-like phages in fact clustered into two different groups. Interestingly, all the phages extracted and purified from the insect extracts shared 99% of their 22-mers, suggesting the prevalence of a single phage species represented by FC03-Usme. BLASTn genome alignments revealed that FC03-Usme, FC30-Tabio, FC28-Sopo and FC39-Tenjo share more than 91.7% sequence identity over 88% coverage, suggesting at least their inclusion in the same genus in a taxonomic sense (this topic will be discussed later). Using shared *k*-mers, we gained additional information on their nucleotide sequence relationships within this group: FC03-Usme only shares 25% of 22-mers long *k*-mers with FC28-Sopo, FC30-Tabio and FC39-Tenjo, whereas it shares closer similarities with FC28-Sopo, FC30-Tabio and FC39-Tenjo. These results suggest that subdivisions exist within the Usme-like phages. Interestingly, FC28-Sopo and FC30-Tabio shared 95% of *k*-mers, while FC28-Sopo and FC30-Tabio only shared 75% of *k*-mers with FC39-Tenjo, separating these three phages into two subgroups represented by FC28-Sopo and FC30-Tabio on one hand, and FC39-Tenjo on the other. This suggests that we can discriminate three variants of the FC03-Usme-like phages using shared *k*-mers. Finally, FC23-Cota, FC41-Suba and FC44-Bosa did not group with each other, nor with any other phages (Figure 2).

### 3.3. Genomic Characterization

To compare our isolated phage genomes with the diversity available in the Genebank database, we performed a nucleotide BLASTn megablast search, optimized to retrieve highly similar sequences using the complete genomic sequences of phages FC03-Usme, FC12-Bacata, FC23-Cota, FC28-Sopo, FC30-Tabio, FC39-Tenjo, FC41-Suba and FC44-Bosa. FC03-Usme, FC12-Bacata and FC44-Bosa shared homology with sequences available in the Genbank database. FC03-Usme displayed high identity with the genome of phage CP2 of *Xanthomonas axonopodis* pv. *citri* [47] (92% identity over 83% coverage), as did FC44-Bosa with phage DLP4 of *Stenotrophomonas maltophilia* [48] (97.4% identity over 99% coverage), and FC12-Bacata with the *Xff*-infecting phage Salvo [28] (96.3% identity over 89% coverage). Two groups represented by phages FC23-Cota and FC41-Suba did not get a hit in any of the phage sequences in the Genbank database, indicating they were indeed totally new.

The general characteristics of the 14 sequenced phage genomes are summarized in Table 4. The genomic organization and the main functions encoded were common to each of the PCR groups defined earlier and are described in Figure S1. FC03-Usme, FC28-Sopo, FC30-Tabio and FC39-Tenjo encoded 58 predicted CDS, with a genome size close to 43 kb and a GC content around 67%. This group shared homology with the CP2 phage genome but to different extents (Figure S2). FC03-Usme shared 92% identity over 83% of the sequence length with the CP2 phage genomic sequence. At the DNA level, FC28-Sopo, FC30-Tabio, and FC39-Tenjo phage genomes showed more than 8% DNA sequence divergence compared to FC03-Usme, but less than 6% compared to the CP2 genome sequence (Figure S2). Interestingly, phages in this group were isolated from different environmental samples collected in different geographical areas such as Florida and Argentina [49], whereas the original CP2 isolate originated from Yokohama, Japan [47]. Moreover, at the DNA level, these phage genomes grouped together and displayed high identity levels, around 93–95% over 83% coverage, confirming the *k*-mer analysis. Finally, FC12-Bacata displayed a genome size of 56 kb with 73 predicted CDS, and is homologous to phage Salvo, with 96% identity and 89% coverage. FC12-Bacata phage is an interesting candidate due to its genomic proximity with a phage that was characterized as active on *Xff* [12].

Surprisingly, the FC44-Bosa phage genome displayed a high identity with the phage DLP4 genome isolated from a soil sample and active on an unrelated species, *Stenotrophomonas maltophilia* [14]. With a genome size of 63 kb and 81 predicted CDS by RAST [38], FC44-Bosa possessed the largest genome of the phages isolated during this study. FC44-Bosa phage was isolated from sewage extracts without any enrichment step, but it seems that FC44-Bosa-like phages are also present in sewage extracts enriched on *Xff* and on insect extract enriched on *Xa*, according to PCR screening (Table 3). A putative integrase gene (CAA2409933.1) has been predicted in FC44-Bosa. A similar gene in the DLP4 genome was confirmed to be involved in the lysogenic infection of *S. maltophilia* D1585 [48]. FC23-Cota phage is the only isolate found in sewage water samples enriched on *Xfm*. Its genome sequence is 42 kb long, and 52 CDS were predicted. This phage genome displayed a low GC content of around 58%, and shared homology was restricted to only 2% of the genome sequence with the *X. citri* phage Xc10 genome [15]. This 2% shared DNA region corresponded to region coding for a DNA polymerase A (ASZ72017.1) and a DNA-dependent RNA polymerase (ASZ72029.1). Finally, the FC41-Suba genome was 45 kb long and 71 CDS were predicted, with the lowest GC content of 52%. As for FC23-Cota, the FC41-Suba genome sequence did not show any homology at the DNA level within the current Genebank database, sharing only a partial sequence of 253 nucleotides with the *A. xylosoxidans* phage phiAxp-1 that corresponds to a region encoding for the Terminase large subunit (YP_009220341.1). The FC23-Cota and FC41-Suba phages were thus totally new and uncharacterized phages.

### 3.4. Morphological Characterization

**Figure 3:**
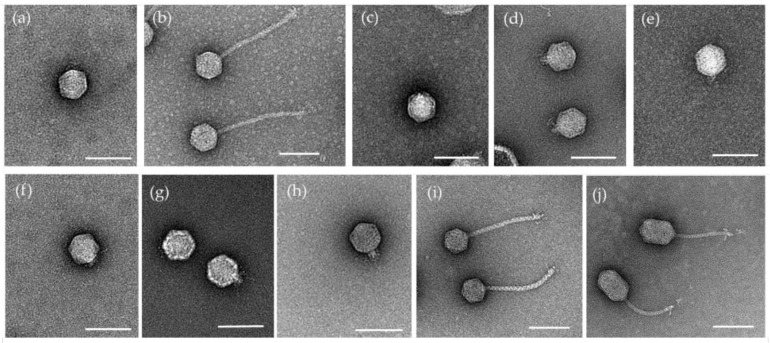
Electron micrographs of isolated phages. Electron micrographs of isolated phages revealed after negative coloration with uranyl acetate 2%. Morphology indicates that the phages belong to two different morphotypes. FC03-Usme (**a**), FC23-Cota (**c**), FC24-Teja (**d**), FC28-Sopo (**e**), FC30-Tabio (**f**), FC34-Cajica (**g**) and FC39-Tenjo (**h**) belong to the podovirus morphotype. FC12-Bacata (**b**), FC41-Suba (**i**) and FC44-Bosa (**j**) belong to the siphovirus morphotype. Scale bar = 100 nm.

To go further with the characterization of all these newly isolated phages, we performed a morphological characterization using Transmission Electronic Microscopy (TEM) after negative staining of the purified particles of FC03-Usme, FC12-Bacata, FC23-Cota, FC24, FC34-Cajica, FC28-Sopo, FC30-Tabio FC39-Tenjo, FC41-Suba and FC44-Bosa. Electron micrographs revealed three morphotypes. FC03-Usme, FC23-Cota, FC24, F32-Sibate, FC28-Sopo, FC30-Tabio and FC39-Tenjo phages showed a typical podophage morphology, whereas FC12-Bacata, FC41-Suba and FC44-Bosa displayed a siphophage morphology (Figure 3). Of note, FC44-Bosa did not display the classical icosahedric capsid morphology, but rather, an elongated one.

Using ImageJ software for image metrics, we determined that CP2-like phages, represented by the podophages FC03-Usme, FC28-Sopo, FC30-Tabio and FC39-Tenjo, displayed an isometric capsid of 61.7 nm, 65.4 nm, 63.6 nm and 60.6 nm, respectively. FC23-Cota, FC24 and FC34-Cajica also exhibit a podophage morphology, with an isometric capsid measuring 64.1 nm, 65.3 nm and 64.4 nm, respectively. Tails and tail fibers were observed on the micrographs, but given their small sizes, the resolution was not sufficient to measure them (Table 5).

In contrast, FC12-Bacata, FC41-Suba and FC44-Bosa showed a siphophage morphology, with two different capsid organizations. FC12-Bacata was composed of an isometric head, the diameter of which was 66.5 nm, and a long and flexible tail of 220.5 nm long. FC41-Suba also had a long and flexible tail of 199.5 nm long, with an isometric capsid of 61.2 nm. In contrast, FC44-Bosa was composed of a heterometric head of 60.1 × 88.8 nm long and a long flexible tail of 140.5 nm. All three siphophages in this study displayed fibers observable at the distal extremity of the tails.

### 3.5. Phylogenetic Analysis of the Sequenced Phage Genomes

Among our PCR groups, we distinguished various degrees of homology with known phage DNA sequences. Phages FC03-Usme, FC12-Bacata and FC44-Bosa could thus be related to phage genomes present in the NCBI database. In contrast, FC23-Cota and FC41-Suba genomes had no substantial sequence similarity throughout the NCBI database (accessed on January 2021).

**Figure 4:**
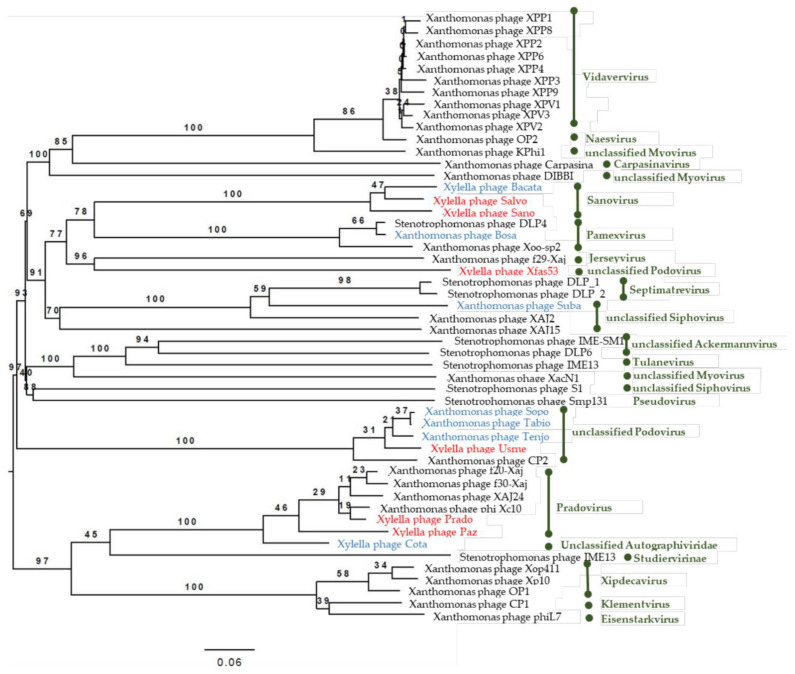
Phylogeny of phages infecting Xanthomonads. The phylogenic tree in Figure 4 shows the relationships between amino acid sequences for the whole phage genomes obtained in this study and the isolated *Caudovirales* phages active on various species of the genera *Xanthomonas*, *Xylella* and *Stenotrophomonas*. All pairwise comparisons of the amino acid sequences were conducted using the Genome-BLAST Distance Phylogeny (GBDP), and trees were inferred using the formulas D0 and yielding average support of 58% method, under the settings recommended for prokaryotic viruses [40]. The OPTSIL clustering yielded 38 species clusters, with 18 at the genus level. Red font highlights referenced bacteriophages that are active on *X. fastidiosa* and blue font highlights referenced bacteriophages isolated in this study. The green font indicates the genus of each phage. Trees were rooted at the midpoint [51]. The numbers above the branches are GBDP pseudo-bootstrap support values from 100 replications. The branch lengths of the resulting VICTOR trees are scaled in terms of the respective distance formula used.

To analyze the phylogenetic relationships of the phages isolated in this study with other phages with lytic activity on *Xanthomonas* spp., *Stenotrophomonas* spp., and *X. fastidiosa*, a phylogenetic tree was generated using the VICTOR online tool [40] using complete genomic sequences of the *Caudovirales* bacteriophages infecting *Xanthomonadales* (Figure 4).

The inferred phylogeny suggested that phages active on the genera *Xanthomonas* and *Stenotrophomonas* were closely related. The OPTSIL clustering yielded 38 species clusters, with 18 at the genus level (Figure 4). In this phylogeny, FC03-Usme, FC28-Sopo, FC30-Tabio and FC39-Tenjo were grouped with phage CP2, and phage FC12-Bacata clustered with *Xff* phages Sano and Salvo. Phage FC23-Cota grouped with the *Xff* phages Prado and Paz [12], and surprisingly, also with *X. arboricola* pv. *juglandis* phages XAJ24, f20-Xaj and f30-Xaj and *X. citri* phage Xc10, a vicinity never described so far. Intriguingly, FC41-Suba remotely clustered with a group containing *Stenotrophonas* phages DLP1 and DLP2, as well as with *X. arboricola* pv. *juglandis* phage XAJ2. Finally, the isolated phage FC44-Bosa clustered with *Stenotrophonas* phage DLP4 and *X. oryzae* pv. *oryzae* Xoo-sp2. Interestingly, this phylogeny, based on complete genomic sequences, was congruent overall with a phylogeny based on the concatenated alignment of the conserved major capsid protein and the terminase large subunit protein, determined from the same phage genomes (Figure S3 and Table S3).

According to the genomic and phylogenic analysis, we propose the following taxonomic description of the viruses isolated in this study according to the latest recommendations [52]. All of them belong to the following lineage: *Viruses*, *Duplodnaviria* (realm), *Heunggongvirae* (kingdom), *Uroviricota* (phylum), *Caudoviricetes* (class), *Caudovirales* (order). Family, sub-family and genus affiliations for the newly isolated viruses are discussed below.

*i:Xylella* phage Cota (*Autographiviridae* family). According to the latest ICTV viral taxonomy criteria (2019 release), the *Xylella* phage Cota belongs to the *Autographiviridae* family. Within this subfamily, the average genome size is 41 kb (42.3 kb for Cota), and the overall genomic organization is conserved with genes on the same strand (predicted ORFs are all situated on the same strand for Cota, Figure S1). *Autographiviridae* virions display a small (ca. 60 nm in diameter) icosahedral head attached to a short tail (podovirus morphology) as is the case for Cota (Figure 3c). Finally, *Autographiviridae* encode their own RNA polymerase, which is the case for Cota (Genbank protein ID CAB1282939.1). A genome-wide phylogenetic analysis based on protein-clustering indicates that *Xylella* phage Cota is related to the *Pradovirus* genus (Figure 4) but classified by OPTSIL as an “Unclassified *Autographiviridae*”. A BLASTn analysis revealed that the two *Pradoviruses Xylella* phage Prado (type species of the *Pradovirus* genus) and *Xylella* phage Paz are almost as distantly related from the DNA point of view (73.08% identity, 27% cover) than *Xylella* phage Prado and *Xylella* phage Cota (68.15% identity and 35% cover). Taking also into account the low pseudo-bootstrap support values, we chose to classify *Xylella* phage Cota within the *Pradovirus* genus (*Autographiviridae* family) and not in a separated new genus as suggested by the phylogenetic tree in Figure 4.

*ii:Usmevirus* genus (*Podoviridae* family). In Figure 4, the genome-wide phylogenetic analysis, based on protein clustering, indicates that *Xylella* phage Usme, *Xanthomonas* phage Sopo, *Xanthomonas* phage Tabio, *Xanthomonas* phage Tenjo (all 4 phages were isolated during this study) and the *Xanthomonas citri* phage CP2 form a new genus within the phages infecting Xanthomonads. *Xylella* phage Usme, *Xanthomonas* phage Sopo, *Xanthomonas* phage Tabio, and *Xanthomonas* phage Tenjo clearly belong to the same species according to the BLASTn analysis (>98.8% identity, >94% cover in pairwise alignments). *Xanthomonas citri* phage CP2 is slightly more distant from the above-cited viruses (identity between 92 to 95%, cover around 83%). Altogether, these results prompt us to classify these five phages within a new genus named “*Usmevirus*” in the *Podoviridae* family. The *Xylella* phage Usme is defined as the type species of this new genus. A proposal will be submitted to the ICTV for the creation of the *Usmevirus* genus. Six other *Xanthomonas* phages were isolated during this study, whose DNA sequences are highly identical to *Xylella* phage Usme according to our *k*-mer analysis (Figure 2). Thus, the *Xanthomonas* phages FC08-Olaya, FC15-Bolivar, FC17-Usaquen, FC25-Alcala, FC47-Fontebon and FC57-Sumapaz all belong to the new *Usmevirus* genus.

*iii:Subavirus* genus (*Siphoviridae* family). In Figure 4, *Xanthomonas* phage FC41-Suba is too distantly related to the *Stenotrophomonas* phages DLP_1 and DLP_2 to be classified in the same *Septimatrevirus* genus (*Siphoviridae* family). We chose to define a new genus named “*Subavirus*” (*Siphoviridae* family) comprising only one *Xanthomonas* phage Suba as a type species for this new genus. A proposal will be submitted to the ICTV for the creation of the *Subavirus* genus.

*iv:Xylella* phage FC12-Bacata. According to the phylogenetic tree presented in Figure 4, the *Xylella* phage Bacata belongs to the *Sanovirus* genus (*Siphoviridae* family).

### 3.6. Host Range Determination

**Figure 5:**
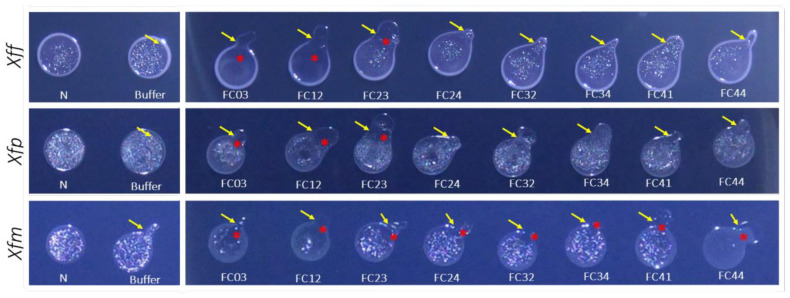
Phage sensitivity spot assay on various *Xf* strains treated with isolated phages. Three *X. fastidiosa* strains were tested. Top line, *X. fastidiosa* subsp. *fastidiosa* (*Xff*) strain CFBP7970, middle line, *X. fastidiosa* subsp. *multiplex* (*Xfm*) strain CFBP 8418, and bottom line, *X. fastidiosa* subsp. *pauca* (*Xfp*) strain CFBP 8402. Aliquots (20 μL) of bacterial suspension were dropped on B-CYE plates and left to dry. Then, 10 μL of FC03-Usme, FC12-Bacata, FC23-Cota, FC24-Tenjo, FC32-Sibate, FC34-Cajica, FC41-Suba, and FC44-Bosa phage suspensions (10^10^ PFU·mL^−1^) were spotted on the margins of the culture drops. Two controls were included for each culture line; N stands for no treatment and B stands for 10 μL of phage buffer. Representative images from three independent experiments are shown. Yellow arrows indicate the inoculation points. Red stars indicate the lytic zones.

To determine the host range of the newly isolated phages on different *Xf* strains, we performed a spot assay using three different bacterial strains representing the subspecies *fastidiosa* (*Xff*), *pauca* (*Xfp*) and *multiplex* (*Xfm*). This assay was performed for the sequenced phages FC03-Usme, FC12-Bacata, FC23-Cota, FC41-Suba and FC44-Bosa. We also included FC24-Teja, isolated from sewage samples enriched on *Xfm* cultures, as well as FC32-Sibate and FC34-Cajica, isolated on *Xa* from runoff waters and without any enrichment on *Xf*. These later phages were selected as potential *Xf* phages, although they were not sequenced (Figure 5).

*Xff* grew faster than *Xfp* and *Xfm*. Thus, for this assay, the *Xff* suspension was adjusted to an OD_600_ of 0.01, whereas *Xfp* and *Xfm* suspensions were adjusted at an OD_600_ of 0.1. *Xff* presents a mucoid phenotype and at the late stages of growth, some crystal-like structures were observed at the surface of the drops. The application of the phage buffer seemed to favor crystal formation at the surface of the drops in all strains. However, clear lysis zones appeared on the *Xff* drops treated with FC03-Usme and FC12-Bacata. A less obvious effect was observed on the *Xff* drop treated with the phage FC23-Cota. In contrast, no lysis zone was observed upon interaction with all other tested phages of that strain. In the *Xfp* strain, the mucoid phenotype was less obvious, but the accumulation of crystals on the drop surface was more significant than with the *Xff* strain. With this strain, we observed a lysis area at the margins of the drops treated with FC03-Usme, FC12-Bacata and FC23-Cota phages, whereas no lytic phenotype could be detected with the other phages. On *Xfm* strain, the drop phenotype was similar to that observed with *Xfp*. *Xfm* was altogether the most sensitive strain to phages, and FC03-Usme and FC12-Bacata were very efficient on this strain. Of note, the application of phages FC03-Usme and FC12-Bacata prevented the formation of crystals on the drop surface. On the *Xfm* strain treated with FC23-Cota, FC24-Teja, FC32-Sibate, FC34-Cajica and FC41-Suba, a small degree of growth inhibition was observed at the interaction points where phages were dropped. Remarkably, even if no lytic zone was observed on *Xfm* in the presence of FC44-Bosa, we observed that this phage affected the phenotype of crystal formation on the drop surface, and the drop also became mucoid. Given the high phage concentration applied, we cannot totally exclude a “lysis from without” phenomenon [53]. However, since similar doses of highly related phages with similar lysis potential from outside were applied with no effect, the observed lysis was likely due to productive phage growth.

We thus found that FC03-Usme, FC12-Bacata and FC23-Cota had lytic activity on the three tested *X. fastidiosa* strains. Moreover, the phages FC24-Teja, FC32-Sibate, FC34-Cajica and FC41-Suba may also have some activity on the *Xfm* strain. The isolated phages presented homology with other bacteriophages infecting bacterial strains belonging to the *Xanthomonaceae* family. Based on the recent phylogeny of the genus *Xanthomonas* genus [54], we selected five other bacterial strains representing different clades, including *X. arboricola* pv. *juglandis* (*Xaj*) for “Clade A”, *X. citri* pv. *citri* (*Xc.ci*) for “Clade B”, *X. vesicatoria* (*Xv*) for “Clade C” and *X. campestris* pv. *campestris* (*Xcc*) for “Clade D” all in the second group of the phylogeny. Another bacterial strain belonging to group one of this tree, *X. translucens* pv. *cerealis* (*Xtc*), was included. As a positive control we used *X. albilineans* (*Xa*). As for *X. fastidiosa* strains, we looked for the lytic ability of isolated bacteriophages using a drop test assay. Only the *Xaj* strain proved sensitive to phage FC12-Bacata (Table 6), in which we did not see very clear lytic activity, but FC12-Bacata seems to slow down *Xaj* growth.

## 4. Discussion

The present work describes the isolation and preliminary characterization of several bacteriophages propagated on *X. albilineans*, including three of them able to infect *X. fastidiosa* subspecies *fastidiosa*, *multiplex* and *pauca*. We focused on these three subspecies, as they were all detected in the Mediterranean area and are thus considered emerging plant pathogens and are included in the European quarantine list. To our knowledge, phages infecting *Xf* were only identified by two different research groups worldwide. The first report mentioning phage particles associated with *Xf* culture was published in 2008 [55]. This study was most likely observing the spontaneous induction of different *Xf* Temecula prophages from cells grown in PW broth, and none of these phage genomes has been isolated or sequenced so far. The same group also documented and analyzed several prophages and their importance in *Xf* strain differentiation [56]. On the other hand, C. Gonzalez’s group isolated and described the first four virulent phages able to infect *Xff* Temecula [28]. In this study, two siphophages, Sano and Salvo, and two podophages, Prado and Paz, were described and their genomic structure analyzed. Importantly, all these phages not only infect several *Xf* strains (including seven *Xff* isolates, two *Xf sandyi* and one *Xf multiplex*), but also the *Xanthomonas* sp. rice isolate (ATCC PTA-13101) that was used as a surrogate host. Prado is the only phage able to infect all 10 *Xf* strains and 6 *Xanthomonas* tested, and thus exhibits a fairly large host range in the *Xanthomonaceae*.

In the present study, we decided to focus on *Xf* isolates that are present in the Mediterranean area, such as *Xfp* CFBP 8402 and *Xfm* CFBP 8418, isolated in the Lecce region in Italy and on the island of Corsica, France, respectively. These two strains are even more fastidious to grow than the type strain *Xff* CFBP 7970, and despite extensive efforts we did not manage to grow them in a soft-agar overlay. For this reason, we decided to use the surrogate host *X. albilineans* for plaquing and production, after enrichment or not on *Xfm* and *Xff* (Figure 1). A striking observation is that phages isolated directly on *Xa* with or without enrichment on *Xa* (FC28-Sopo, FC30-Tabio, FC39-Tenjo, FC41-Suba, FC44-Bosa) were not able to infect *Xff* and *Xfp* strains but could infect *Xfm* with a low activity. In contrast, the three phages isolated upon enrichment on *Xfm* or *Xff* were able to infect efficiently all three *Xf* strains included in our spot assay (Figure 5, Table 6). This highlights the absolute requirement of the enrichment step in order to isolate phages active against *Xf*.

Regarding the isolation sources and strategy, we could not isolate any phage from plant samples displaying the typical symptoms of *Xf*-infected plants and collected in an infected area of Corsica. Of note, virulent phages able to infect *Xff* were isolated from plant extracts (rice and weeds) that contained *Xanthomonas* spp. but not *Xf* [28]. Different hypotheses could explain this result: (i) the processing of the plant samples (see Section 2.2) could have been detrimental to phage isolation, (ii) the symptoms observed on the collected plant samples were not due to *Xf* infection, or (iii) the symptoms were indeed due to the presence of *Xf*, but specific phages did not develop in these plants. In contrast, insect vector samples harvested on the island of Corsica, close to the plant samples, were processed in a very similar way to the plant extracts and proved to be an actual phage reservoir for phage FC03-Usme and relatives belonging to the CP2-like group (Table 4). Although different phage isolates were obtained from this type of biological sample, they all proved to belong to the same group. To our knowledge, this is the first example of a virulent phage, active against *Xf*, isolated from such a source. Interestingly, the xylem-feeding leafhopper vector, called the glassy-winged sharpshooter (GWSS, *Homalodisca* genus, Hemiptera: Cicadellidae), which is the main insect vector identified for Pierce’s disease transmission [57], has the ability to uptake phages. In this experiment, the insect vector was allowed to feed on beam stems immersed in a phage Paz solution, and showed efficient acquisition of that phage up to 10^8^ PFU gm^−1^ of GWSS tissue [58]. In the same work, however, the authors mentioned that although phage Paz was efficiently acquired by GWSS, its transmission to plants (bean stems) through the same insect vector was much less efficient, probably due to a dilution effect. It is thus remarkable that several phages were successfully isolated from insect extracts collected in the *Xf*-infected area. The environmental source that provided the largest diversity in terms of phage isolates was the sewage water sample collected in the Marseille wastewater plant. This is not surprising, as worldwide phage researchers successfully isolate bacteriophages from sewage samples, targeting an immense variety of human, plant, or animal pathogens [59]. The particularity of the water treatment plant we had access to is that it collects water not only from Marseille, the largest city in the region, but also from many other cities and villages around the area with strong agricultural activity. This may explain the significant diversity of phages that could be isolated from this source.

The genomic analysis of the newly isolated phages led to interesting observations. First of all, we isolated and sequenced four different phages that all share a high genomic identity with *X. axonopodis* phage CP2, among which only FC03-Usme was able to infect *Xf* strains. This suggest that the CP2-like phages, whose first isolate came from Yokohama (Japan), are very abundant and are distributed all over the world, probably with the *Xanthomonaceae* strains they infect. A similar conclusion applies to the phage FC12-Bacata, whose genome sequence is homologous to that of the phage Salvo isolated in Texas (USA). Then come phage genomes with very low identity to any genome present in the databases, such as FC23-Cota and FC41-Suba, which can be considered new phage species since their genomes only match a few conserved genes in the *X. citri* phage Xc10 and *A. xylosoxidans* phage phiAxp-1 genomes, respectively. Finally, the procedure we set up in this study led mostly to the isolation of strictly virulent phages, as no specific signature of lysogenic development could be identified in their genomes, such as integrase or repressor genes; the BLAST search did not reveal any homology with *Xanthomonaceae* strains genomes so effectively, as temperate phage genomes would do. The only temperate phage isolated was named FC44-Bosa, whose genome shared extensive identity with the *Stenotrophomonas* phage DLP4 (Table 4). Interestingly, this phage showed good lytic activity on *Xfm*.

## 5. Conclusions

This study allowed the isolation of several phages that constitute promising candidates for the biocontrol of *Xylella* species in planta. However, in order to reach this goal, additional experimental work is needed. In particular, this project will require the setting up of plant assays to assess phage dissemination, as well as efficient *Xf* eradication in planta. The application strategy also needs to be carefully designed, since *Xf* is restricted to plant xylem and application modes designed for other plant pathogens might be ineffective. Current application strategies encompass seed treatment, post-harvest spraying, soil-based or watering system delivery, and plant puncturing [60]. The choice of the best delivery system should be thus performed in agreement with the lifestyle of the bacterium and tested experimentally.

## Figures and Tables

**Table 1:** Bacterial strains used in this study.

Strain/Genotype	Abbreviation	Collection Code	NCBI
*Xylella fastidiosa* subsp. *fastidiosa*	*Xff*	CFBP 7970	PRJNA417585
*Xylella fastidiosa* subsp. *pauca*	*Xfp*	CFBP 8402	PRJNA383475
*Xylella fastidiosa* subsp. *multiplex*	*Xfm*	CFBP 8418	PRJNA314986
*Xanthomonas albilineans*	*Xa*	CFBP 2523	PRJNA338244
*Xanthomonas arboricola* pv. *juglandis*	*Xaj*	CFBP 2528	PRJNA275685
*Xanthomonas citri* pv. *citri*	*Xcci*	CFBP 3369	PRJNA254373
*Xanthomonas vesicatoria*	*Xv*	CFBP 2537	PRJNA60019
*Xanthomonas campestris* pv. *campestris*	*Xcc*	CFBP 5241	PRJNA296
*Xanthomonas translucens* pv. *cerealis*	*Xtc*	CFBP 2541	PRJNA268946

**Table 2:** Phage genomes as sequenced.

Phage Name	Full Nomenclature	Accession Number	Raw Sequence ID
FC03-Usme FC08-Olaya	*Xylella* phage Usme *Xanthomonas* phage Olaya	LR743523 MW802488	ERR5548206 SRX10492030
FC12-Bacata FC15-Bolivar FC17-Usaquen	*Xylella* phage Bacata *Xanthomonas* phage Bolivar *Xanthomonas* phage Usaquen	LR743524 MW822535 MW822536	ERR5548205 SRX10492031 SRX10492032
FC23-Cota FC25-Alcala	*Xylella* phage Cota *Xanthomonas* phage Alcala	LR778216 MW822537	ERR5548199 SRX10492033
FC28-Sopo	*Xanthomonas* phage Sopo	LR743529	ERR5548204
FC30-Tabio	*Xanthomonas* phage Tabio	LR743528	ERR5548203
FC39-Tenjo	*Xanthomonas* phage Tenjo	LR743531	ERR5548202
FC41-Suba	*Xanthomonas* phage Suba	LR743530	ERR5548201
FC44-Bosa FC47-Fontebon FC57-Sumapaz	*Xanthomonas* phage Bosa *Xanthomonas* phage Fontebon *Xanthomonas* phage Sumapaz	LR743532 MW822538 MW822539	ERR5548200 SRX10492034 SRX10492035

**Table 3:** Phages isolated with and without enrichment.

				PCR Groups ^a^				
Source	Conditions	Isolated Phages	Sequenced Phage Genomes	FC03	FC44	FC12	FC23	Potential New Phages
Plant extracts	Enrichment on *Xff*	NA ^b^	-	-	-	-	-	-
	Enrichment on *Xfm*	NA	-	-	-	-	-	-
	Enrichment on *Xa*	NA	-	-	-	-	-	-
Insect extracts	Enrichment on *Xff*	6	LR743523 MW802488	6	-	-	-	0
	Enrichment on *Xfm*	6	MW822538 MW822539	6	-	-	-	0
	Enrichment on *Xa*	12	MW822535 MW822536	11	1	-	-	0
Sewage watersample	Enrichment on *Xff*	6	LR743524 (x2) ^c^	-	2	4	-	0
	Enrichment on *Xfm*	6	LR778216 MW822537	1	1	-	3	1
	Direct plaquing on *Xa*	12	LR743531 LR743532	4	4	2	-	1 + LR743530 ^d^
Runoff waters sample	Enrichment on *Xff*	NA	-	-	-	-	-	-
	Enrichment on *Xfm*	NA	-	-	-	-	-	-
	Direct plaquing on *Xa*	8	LR743529 LR743528	4	-	-	-	4

^a^ PCR groups using specific primers targeting the isolated and sequenced phage genomes. ^b^ NA, not applicable. ^c^ LR743524 has been sequenced twice. ^d^ LR743530 was selected after PCR screening.

**Table 4:** General features of 14 sequenced phage genomes. In this table, we temporarily assign our newly sequenced phages the taxonomic affiliation of their closest homologs (when available).

Phage	Size (bp)	%GC	CDS	BLAST Nearest Organism	Coverage (%)	Identity (%)	E-Value	Order	Family	Genus	Specie
FC03-Usme ^a^	43721	66.6	58	*X. axonopodis* phage CP2	83	92.11%	0.0	Caudovirales	Podovirus	Unclassified Podovirus	Usmevirus
FC12-Bacata	56232	62.9	73	*X. fastidiosa* phage Salvo	89	96.29%	0.0	Caudovirales	Siphovirus	Sanovirus	Bacatavirus
FC23-Cota	42307	58.8	52	none	NA	NA	NA	NA	NA	NA	Tejovirus
FC28-Sopo	43861	66.9	58	*X. axonopodis* phage CP2	82	93.96%	0.0	Caudovirales	Podovirus	Unclassified Podovirus	Bacatavirus
FC30-Tabio	43458	66.9	58	*X. axonopodis* phage CP2	83	95.39%	0.0	Caudovirales	Podovirus	Unclassified Podovirus	Bacatavirus
FC39-Tenjo	43850	67.2	58	*X. axonopodis* phage CP2	83	94.70%	0.0	Caudovirales	Podovirus	Unclassified Podovirus	Bacatavirus
FC41-Suba	45510	52.3	71	none	NA	NA	NA	NA	NA	NA	Subavirus
FC44-Bosa	63828	64.8	81	*Stenotrophomonas* phage DLP4	99%	97.40 %	0.0	Caudovirales	Siphovirus	Unclassified Siphovirus	Bosavirus

^a^ FC08-Olaya, FC15-Bolivar, FC-17 Usaquen, FC25-Alcala, FC47-Fontebon and FC57-Sumapaz genomes have the same characteristics as FC03-Usme.

**Table 5:** Morphological characteristics of the phages based on MET acquisitions.

Phage	Head Shape & Diameter (nm)	Tail Shape & Length (nm)	Morphotype ^a^	
FC03-Usme (*n* = 15)	Isometric, 61.7 ± 2	*NA	Podovirus	C1
FC12-Bacata (*n* = 14)	Isometric, 66.5 ± 2	Long, Flexible, 220.5 ± 5	Siphovirus	B1
FC23-Cota (*n* = 14)	Isometric, 64.1 ± 1	*NA	Podovirus	C1
FC24-Teja (*n* = 15)	Isometric, 65.3 ± 2	*NA	Podovirus	C1
FC28-Sopo (*n* = 6)	Isometric, 65.4 ± 2	*NA	Podovirus	C1
FC30-Tabio (*n* = 6)	Isometric, 63.6 ± 2	*NA	Podovirus	C1
FC34-Cajica (*n* = 15)	Isometric, 64.4 ± 2	*NA	Podovirus	C1
FC39-Tenjo (*n* = 6)	Isometric, 60.6 ± 2	*NA	Podovirus	C1
FC41-Suba (*n* = 15)	Isometric, 61.2 ± 2	Long, Flexible, 199.5 ± 4	Siphovirus	B1
FC44-Bosa (*n* = 12)	Heterometric, 60.1 ± 1 × 88.8 ± 2	Long, Flexible, 140.5 ± 7	Siphovirus	B3

Phage physical dimensions are the means of measurements of at least *n* individual virions using ImageJ. *NA, not applicable; ^a^ according to [50].

**Table 6:** Lytic activity of phages on *Xylella* and *Xanthomonas* species.

CFBP	Strain	FC03-Usme	FC12-Bacata	FC23-Cota	FC24-Teja	FC32-Tabio	FC34-Tenjo	FC41-Suba	FC44-Bosa
7970	*X. fastidiosa* subsp. *fastidiosa*								
8402	*X. fastidiosa* subsp. *pauca*								
8418	*X. fastidiosa* subsp. *multiplex*								
2523	*X. albilineans*								
2528	*X. arboricola* pv. *juglandis*								
3369	*X. citri* pv. *citri*								
2537	*X. vesicatoria*								
5241	*X. campestris* pv. *campestris*								
2541	*X. translucens* pv. *cerealis*								

Black boxes indicate a lytic activity of the selected phages, gray boxes represent a weak lytic activity, and white boxes indicate no lytic activity.

## Data Availability

The data presented in this study are openly available in European Nucleotide Archive (project PRJEB35136) and NCBI (see Table 2 for detailed accession numbers).

## Supplementary Materials

The following are available online at https://www.mdpi.com/article/10.3390/v13050725/s1, Figure S1: Genomic organization and main functions encoded by FC03-Usme, FC12-Bacata, FC23-Cota, FC41-Suba and FC44-Bosa phage genomes, the type phages of the 5 PCR groups defined in this study, Figure S2: Visualization of BLAST-pairwise comparisons of genome sequences of FCO3-Usme-like phages and *Xanthomonas* ax-onopodis phage CP2, Figure S3: Phylogeny of phages infecting *Xanthomonads*, Table S1: Genomic information used for whole genome phylogeny in Figure 4, Table S2: Comparison matrix for similarities between genomes of the phages sequenced in this study, Table S3: Genomic information used for MCP and Terminase_lsu phylogeny.

## References

[1] Jeger M., Caffier D., Candresse T., Chatzivassiliou E., Dehnen-Schmutz K., Gilioli G., Grégoire J.-C., Jaques Miret J.A., MacLeod A., EFSA Panel on Plant Health (EFSA PLH Panel) (2018). Updated Pest Categorisation of Xylella Fastidiosa. EFSA J..

[2] Alston J.M., Fuller K.B., Kaplan J.D., Tumber K.P. (2015). Assessing the Returns to R&D on Perennial Crops: The Costs and Benefits of Pierce’s Disease Research in the California Winegrape Industry. Aust. J. Agric. Resour. Econ..

[3] Tumber K.P., Alston J.M., Fuller K. (2014). Pierce’s Disease Costs California $104 Million per Year. Calif. Agric..

[4] Schneider K., van der Werf W., Cendoya M., Mourits M., Navas-Cortés J.A., Vicent A., Lansink A.O. (2020). Impact of Xylella Fastidiosa Subspecies Pauca in European Olives. PNAS.

[5] Ali B.M., van der Werf W., Oude Lansink A. (2021). Assessment of the Environmental Impacts of Xylella Fastidiosa Subsp. Pauca in Puglia. Crop Prot..

[6] Simpson A.J., Reinach F.C., Arruda P., Abreu F.A., Acencio M., Alvarenga R., Alves L.M., Araya J.E., Baia G.S., Baptista C.S. (2000). The Genome Sequence of the Plant Pathogen Xylella Fastidiosa. The Xylella Fastidiosa Consortium of the Organization for Nucleotide Sequencing and Analysis. Nature.

[7] Falahi Charkhabi N., Booher N.J., Peng Z., Wang L., Rahimian H., Shams-Bakhsh M., Liu Z., Liu S., White F.F., Bogdanove A.J. (2017). Complete Genome Sequencing and Targeted Mutagenesis Reveal Virulence Contributions of Tal2 and Tal4b of Xanthomonas Translucens Pv. Undulosa ICMP11055 in Bacterial Leaf Streak of Wheat. Front. Microbiol..

[8] Pieretti I., Royer M., Barbe V., Carrere S., Koebnik R., Cociancich S., Couloux A., Darrasse A., Gouzy J., Jacques M.-A. (2009). The Complete Genome Sequence of Xanthomonas Albilineans Provides New Insights into the Reductive Genome Evolution of the Xylem-Limited Xanthomonadaceae. BMC Genom..

[9] Gerlin L., Cottret L., Cesbron S., Taghouti G., Jacques M.-A., Genin S., Baroukh C. (2020). Genome-Scale Investigation of the Metabolic Determinants Generating Bacterial Fastidious Growth. mSystems.

[10] Pierce N.B., Newton B. (1892). The California Vine Disease: A Preliminary Report of Investigations.

[11] Davis M.J., Purcell A.H., Thomson S.V. (1978). Pierce’s Disease of Grapevines: Isolation of the Causal Bacterium. Science.

[12] Wells J.M., Raju B.C., Hung H.-Y., Weisburg W.G., Mandelco-Paul L., Brenner D.J. (1987). Xylella Fastidiosa Gen. Nov., Sp. Nov: Gram-Negative, Xylem-Limited, Fastidious Plant Bacteria Related to Xanthomonas Spp.. Int. J. Syst. Evol. Microbiol..

[13] Mansfield J., Genin S., Magori S., Citovsky V., Sriariyanum M., Ronald P., Dow M., Verdier V., Beer S.V., Machado M.A. (2012). Top 10 Plant Pathogenic Bacteria in Molecular Plant Pathology: Top 10 Plant Pathogenic Bacteria. Mol. Plant Pathol..

[14] Jacques M.-A., Arlat M., Boulanger A., Boureau T., Carrère S., Cesbron S., Chen N.W.G., Cociancich S., Darrasse A., Denancé N. (2016). Using Ecology, Physiology, and Genomics to Understand Host Specificity in Xanthomonas. Annu. Rev. Phytopathol..

[15] Bragard C., Dehnen-Schmutz K., Serio F.D., Gonthier P., Jacques M.-A., Miret J.A.J., Justesen A.F., MacLeod A., Magnusson C.S., Milonas P. (2019). Effectiveness of in Planta Control Measures for Xylella Fastidiosa. EFSA J..

[16] Dupas E., Briand M., Jacques M.-A., Cesbron S. (2019). Novel Tetraplex QPCR Assays for Simultaneous Detection and Identification of Xylella Fastidiosa Subspecies in Plant Tissues. bioRxiv.

[17] (2006). Directive européene 2006/7/EC Directive 2006/7/EC of the European Parliament and of the Council of 15 February 2006 Concerning the Management of Bathing Water Quality and Repealing Directive 76/160/EEC. Off. J. Eur. Union.

[18] (2017). Andrea Luvisi; Francesca Nicolì; Luigi De Bellis Sustainable Management of Plant Quarantine Pests: The Case of Olive Quick Decline Syndrome. Sustainability.

[19] (2016). EFSA Panel on Plant Health (PLH) Treatment Solutions to Cure Xylella Fastidiosa Diseased Plants. EFSA J..

[20] Scortichini M., Chen J., Caroli M.D., Dalessandro G., Pucci N., Modesti V., L’aurora A., Petriccione M., Zampella L., Mastrobuoni F. (2018). A Zinc, Copper and Citric Acid Biocomplex Shows Promise for Control of Xylella Fastidiosa Subsp. Pauca in Olive Trees in Apulia Region (Southern Italy). Phytopathol. Mediterr..

[21] Hopkins D.L. (2005). Biological Control of Pierce’s Disease in the Vineyard with Strains of Xylella Fastidiosa Benign to Grapevine. Plant Dis..

[22] Baccari C., Antonova E., Lindow S. (2019). Biological Control of Pierce’s Disease of Grape by an Endophytic Bacterium. Phytopathology.

[23] Vandamme E.J., Mortelmans K. (2019). A Century of Bacteriophage Research and Applications: Impacts on Biotechnology, Health, Ecology and the Economy!: A Century of Bacteriophage Research and Applications. J. Chem. Technol. Biotechnol..

[24] Manyi-Loh C., Mamphweli S., Meyer E., Okoh A. (2018). Antibiotic Use in Agriculture and Its Consequential Resistance in Environmental Sources: Potential Public Health Implications. Molecules.

[25] Żaczek M., Weber-Dąbrowska B., Górski A. (2015). Phages in the Global Fruit and Vegetable Industry. J. Appl. Microbiol..

[26] Sieiro C., Areal-Hermida L., Pichardo-Gallardo Á., Almuiña-González R., de Miguel T., Sánchez S., Sánchez-Pérez Á., Villa T.G. (2020). A Hundred Years of Bacteriophages: Can Phages Replace Antibiotics in Agriculture and Aquaculture?. Antibiotics.

[27] Buttimer C., McAuliffe O., Ross R.P., Hill C., O’Mahony J., Coffey A. (2017). Bacteriophages and Bacterial Plant Diseases. Front. Microbiol..

[28] Ahern S.J., Das M., Bhowmick T.S., Young R., Gonzalez C.F. (2014). Characterization of Novel Virulent Broad-Host-Range Phages of Xylella Fastidiosa and Xanthomonas. J. Bacteriol..

[29] Das M., Bhowmick T.S., Ahern S.J., Young R., Gonzalez C.F. (2015). Control of Pierce’s Disease by Phage. PLoS ONE.

[30] Gonzalez C.F., Ahern S.J., Das M., Young I.R.F., Bhowmick T.S. (2015). Methods and Compositions for Treatment and Control of Plant Disease. U.S. Patent.

[31] (2016). PM 7/24 (2) Xylella Fastidiosa. EPPO Bull..

[32] Lopes S.A., Torres S.C.Z. (2006). An Effective and Low-Cost Culture Medium for Isolation and Growth of Xylella Fastidiosa from Citrus and Coffee Plants. Curr. Microbiol..

[33] Saux M.F.-L., Bonneau S., Essakhi S., Manceau C., Jacques M.-A. (2015). Aggressive Emerging Pathovars of Xanthomonas Arboricola Represent Widespread Epidemic Clones Distinct from Poorly Pathogenic Strains, as Revealed by Multilocus Sequence Typing. Appl. Environ. Microbiol..

[34] La Situation de Xylella en France et en Europe | Alim’agri. http://agriculture.gouv.fr/la-situation-de-xylella-en-france-et-en-europe.

[35] Schneider C.A., Rasband W.S., Eliceiri K.W. (2012). NIH Image to ImageJ: 25 Years of Image Analysis. Nat. Methods.

[36] Li Y., Hu Y., Bolund L., Wang J. (2010). State of the Art de Novo Assembly of Human Genomes from Massively Parallel Sequencing Data. Hum. Genom..

[37] Zerbino D.R., Birney E. (2008). Velvet: Algorithms for de Novo Short Read Assembly Using de Bruijn Graphs. Genome Res..

[38] Aziz R.K., Bartels D., Best A.A., DeJongh M., Disz T., Edwards R.A., Formsma K., Gerdes S., Glass E.M., Kubal M. (2008). The RAST Server: Rapid Annotations Using Subsystems Technology. BMC Genom..

[39] Briand M., Bouzid M., Hunault G., Legeay M., Saux M.F.-L., Barret M. (2019). A Rapid and Simple Method for Assessing and Representing Genome Sequences Relatedness. bioRxiv.

[40] Meier-Kolthoff J.P., Göker M. (2017). VICTOR: Genome-Based Phylogeny and Classification of Prokaryotic Viruses. Bioinformatics.

[41] Göker M., García-Blázquez G., Voglmayr H., Tellería M.T., Martín M.P. (2009). Molecular Taxonomy of Phytopathogenic Fungi: A Case Study in Peronospora. PLoS ONE.

[42] Adriaenssens E., Brister J.R. (2017). How to Name and Classify Your Phage: An Informal Guide. Viruses.

[43] El Haddad L., Ben Abdallah N., Plante P.-L., Dumaresq J., Katsarava R., Labrie S., Corbeil J., St-Gelais D., Moineau S. (2014). Improving the Safety of Staphylococcus Aureus Polyvalent Phages by Their Production on a Staphylococcus Xylosus Strain. PLoS ONE.

[44] González-Menéndez E., Arroyo-López F.N., Martínez B., García P., Garrido-Fernández A., Rodríguez A. (2018). Optimizing Propagation of Staphylococcus Aureus Infecting Bacteriophage VB_SauM-PhiIPLA-RODI on Staphylococcus Xylosus Using Response Surface Methodology. Viruses.

[45] David H.L., Clavel S., Clement F. (1980). Adsorption and Growth of the Bacteriophage D29 in Selected Mycobacteria. Ann. L’institut Pasteur Virol..

[46] Santos S.B., Fernandes E., Carvalho C.M., Sillankorva S., Krylov V.N., Pleteneva E.A., Shaburova O.V., Nicolau A., Ferreira E.C., Azeredo J. (2010). Selection and Characterization of a Multivalent Salmonella Phage and Its Production in a Nonpathogenic Escherichia Coli Strain. Appl. Environ. Microbiol..

[47] Ahmad A.A., Ogawa M., Kawasaki T., Fujie M., Yamada T. (2014). Characterization of Bacteriophages Cp1 and Cp2, the Strain-Typing Agents for Xanthomonas Axonopodis Pv. Citri. Appl. Environ. Microbiol..

[48] Peters D., McCutcheon J., Stothard P., Dennis J. (2019). Novel Stenotrophomonas Maltophilia Temperate Phage DLP4 Is Capable of Lysogenic Conversion. BMC Genom..

[49] Balogh B. (2006). Characterization and Use of Bacteriophages Associated with Citrus Bacterial Pathogens for Disease Control. Ph.D. Thesis.

[50] Ackermann H.W. (2001). Frequency of Morphological Phage Descriptions in the Year 2000. Brief Review. Arch. Virol..

[51] Farris J.S. (1972). Estimating Phylogenetic Trees from Distance Matrices. Am. Nat..

[52] Turner D., Kropinski A.M., Adriaenssens E.M. (2021). A Roadmap for Genome-Based Phage Taxonomy. Viruses.

[53] Abedon S.T. (2011). Lysis from Without. Bacteriophage.

[54] Merda D., Bonneau S., Guimbaud J.-F., Durand K., Brin C., Boureau T., Lemaire C., Jacques M.-A., Fischer-Le Saux M. (2016). Recombination-Prone Bacterial Strains Form a Reservoir from Which Epidemic Clones Emerge in Agroecosystems. Environ. Microbiol. Rep..

[55] Chen J., Civerolo E.L. (2008). Morphological Evidence for Phages in Xylella Fastidiosa. Virol. J..

[56] de Mello Varani A., Souza R.C., Nakaya H.I., de Lima W.C., Paula de Almeida L.G., Kitajima E.W., Chen J., Civerolo E., Vasconcelos A.T.R., Van Sluys M.-A. (2008). Origins of the Xylella Fastidiosa Prophage-like Regions and Their Impact in Genome Differentiation. PLoS ONE.

[57] Almeida R.P.P., Purcell A.H. (2003). Transmission of Xylella Fastidiosa to Grapevines by Homalodisca Coagulata (Hemiptera: Cicadellidae). J. Econ. Entomol..

[58] Bhowmick T.S., Das M., Heinz K.M., Krauter P.C., Gonzalez C.F. (2016). Transmission of Phage by Glassy-Winged Sharpshooters, a Vector of Xylella Fastidiosa. Bacteriophage.

[59] Chanishvili N. (2012). Phage Therapy--History from Twort and d’Herelle through Soviet Experience to Current Approaches. Adv. Virus Res..

[60] Holtappels D., Fortuna K., Lavigne R., Wagemans J. (2021). The Future of Phage Biocontrol in Integrated Plant Protection for Sustainable Crop Production. Curr. Opin. Biotechnol..

