# Supplementary material for: Novel Virulent Bacteriophages Infecting Mediterranean Isolates of the Plant Pest Xylella fastidiosa and Xanthomonas albilineans
Source: Viruses. 2021 Apr 21;13(5):725. doi: 10.3390/v13050725 (PMC8143317; doi:10.3390/v13050725)
Supplement: Supplementary file 1 [file viruses-13-00725-s001.zip › viruses-1158992-SI.pdf]

viruses

Table S1. Genomic information used for whole genome phylogeny in Figure 4.

Phage name	Host	Family	Size (nt)	GenBank	Order	Family	Genus	Subfamily	Specie
Stenotrophomonas phage DLP4	*S. maltophilia* D1585	*Siphoviridae*	63945	MG018224.1	Caudovirales	Siphoviridae	Pamexvirus	unclassified Pamexvirus	
Stenotrophomonas phage IME13	*S. maltophilia*	*Myoviridae*	162327	JX306041.1	Caudovirales	Myoviridae	Tulanevirus		Stenotrophomonas virus IME13
Stenotrophomonas phage IME15	*S. maltophilia*	*Podoviridae*	38513	JX872508.1	Caudovirales	Autographiviridae	Studierivirinae	Teseptimavirus	Stenotrophomonas virus IME15
Stenotrophomonas phage IME-SM1	*S. maltophilia*	*unclassified*	159514	KR560069.1	Caudovirales	Ackermannviridae	unclassified Ackermannviridae		
Stenotrophomonas phage S1	*S. maltophilia*	*Siphoviridae*	40287	EU849489.1	Caudovirales	Siphoviridae	unclassified Siphoviridae		
Stenotrophomonas phage Smp131	*S. maltophilia*	*Myoviridae*	33525	JQ809663.1	Caudovirales	Myoviridae	Peduovirinae	Simpcentumvirus	
Stenotrophomonas phage vB_SmaS-DLP_1	*S. maltophilia* D1585	*Siphoviridae*	42887	KR537872.1	Caudovirales	Siphoviridae	Septimatrevirus	unclassified Septimatrevirus	
Stenotrophomonas phage vB_SmaS-DLP_2	*S. maltophilia* D1585	*Siphoviridae*	42593	KR537871.1	Caudovirales	Siphoviridae	Septimatrevirus	unclassified Septimatrevirus	
Stenotrophomonas phage vB_SmaS-DLP_6	*S. maltophilia* D1571	*Myoviridae*[1]	168489	KU682439.2	Caudovirales	Ackermannviridae	unclassified Ackermannviridae		
Xanthomonas phage Carpasina	*X. campestris*	*Myoviridae*	61939	MH059633.1	Caudovirales	Myoviridae	Carpasinavirus		Xanthomonas virus Carpasina
Xanthomonas phage CP1	*X. axonopodis* pv. *citri*	*Siphoviridae*	43870	AB720063.2	Caudovirales	Siphoviridae	Klementvirus		
Xanthomonas phage CP2	*X. axonopodis* pv. *citri*	*Podoviridae*	42963	AB720064.1	Caudovirales	Podoviridae	unclassified Podoviridae		
Xanthomonas phage f20-Xaj	*X. arboricola* pv. *juglandis* X-J303	*Podoviridae*	43851	NC_030928.1	Caudovirales	Autographiviridae	Pradovirus		Xanthomonas virus f20
Xanthomonas phage f29-Xaj	*X. arboricola* pv.	*Siphoviridae*	41865	KU595434.1	Caudovirales	Siphoviridae	Jerseyvirus	unclassified Jerseyvirus	

	juglandis X-J303								
Xanthomonas phage f30-Xaj	*X. arboricola* pv. *juglandis* X-J303	*Podoviridae*	44262	NC_030937.1	Caudovirales	Autographiviridae	Pradovirus		
Xanthomonas phage KPhi1	*X. euvesicatoria*	*Myoviridae*	46077	KY210139.1	Caudovirales	Myoviridae	unclassified Myoviridae		
Xanthomonas phage OP1	*X. oryzae* pv. *oryzae*	*Siphoviridae*	43785	AP008979.1	Caudovirales	Siphoviridae	Xipdecavirus		
Xanthomonas phage OP2	*X. oryzae* pv. *oryzae*	*Myoviridae*	46643	AP008986.1	Caudovirales	Myoviridae	Naesvirus		Xanthomonas virus OP2
Xanthomonas phage phi Xc10	*X. campestris* pv. *citri*	*Podoviridae*	44597	MF375456.1	Caudovirales	Autographiviridae	Pradovirus		
Xanthomonas phage phiL7	*X. campestris* pv. *campestris*	*Siphoviridae*	44080	EU717894.1	Caudovirales	Siphoviridae	Eisenstarkvirus		
Xanthomonas phage vB_XveM_DIBBI	*X. vesicatoria*	*Myoviridae*	49981	JN022534.1	Caudovirales	Myoviridae	unclassified Myoviridae		
Xanthomonas phage XacN1	*X. citri*	*Myoviridae*	384670	AP018399.1	Caudovirales	Myoviridae	unclassified Myoviridae		
Xanthomonas phage XAJ2	*X. arboricola* pv. *juglandis*	*Siphoviridae*	49241	KU197014.1	Caudovirales	Siphoviridae	unclassified Siphoviridae		
Xanthomonas phage XAJ24	*X. arboricola* pv. *juglandis*	*Podoviridae*	44861	KU197013.1	Caudovirales	Autographiviridae	Pradovirus		
Xanthomonas phage Xoo-sp2	*X. oryzae* pv. *oryzae*	*Siphoviridae*	60497	KX241618.1	Caudovirales	Siphoviridae	Pamexvirus	unclassified Pamexvirus	
Xanthomonas phage Xop411	*X. oryzae*	*Siphoviridae*	44520	DQ777876.1	Caudovirales	Siphoviridae	Xipdecavirus		
Xanthomonas phage Xp10	*X. oryzae*	*Siphoviridae*	44373	AY299121.1	Caudovirales	Siphoviridae	Xipdecavirus		
Xanthomonas phage Xp15	*X. campestris* pv. *pelargonii*	*Siphoviridae*	55770	AY986977.1	Caudovirales	Siphoviridae	unclassified Siphoviridae		
Xanthomonas phage XPP1	*X. oryzae* pv. *oryzae*	*Myoviridae*	46195	MG944227.1	Caudovirales	Myoviridae	Vidavervirus	unclassified Vidavervirus	

Xanthomonas phage XPP2	*X. oryzae* pv. *oryzae*	*Myoviridae*	46480	MG944228.1	Caudovirales	Myoviridae	Vidavervirus	unclassified Vidavervirus
Xanthomonas phage XPP3	*X. oryzae* pv. *oryzae*	*Myoviridae*	49612	MG944229.1	Caudovirales	Myoviridae	Vidavervirus	unclassified Vidavervirus
Xanthomonas phage XPP4	*X. oryzae* pv. *oryzae*	*Myoviridae*	47397	MG944230.1	Caudovirales	Myoviridae	Vidavervirus	unclassified Vidavervirus
Xanthomonas phage XPP6	*X. oryzae* pv. *oryzae*	*Myoviridae*	46281	MG944231.1	Caudovirales	Myoviridae	Vidavervirus	unclassified Vidavervirus
Xanthomonas phage XPP8	*X. oryzae* pv. *oryzae*	*Myoviridae*	46278	MG944232.1	Caudovirales	Myoviridae	Vidavervirus	unclassified Vidavervirus
Xanthomonas phage XPP9	*X. oryzae* pv. *oryzae*	*Myoviridae*	48669	MG944233.1	Caudovirales	Myoviridae	Vidavervirus	unclassified Vidavervirus
Xanthomonas phage XPV1	*X. oryzae* pv. *oryzae*	*Myoviridae*	46503	MG944234.1	Caudovirales	Myoviridae	Vidavervirus	unclassified Vidavervirus
Xanthomonas phage XPV2	*X. oryzae* pv. *oryzae*	*Myoviridae*	45969	MG944235.1	Caudovirales	Myoviridae	Vidavervirus	unclassified Vidavervirus
Xanthomonas phage XPV3	*X. oryzae* pv. *oryzae*	*Myoviridae*	47046	MG944236.1	Caudovirales	Myoviridae	Vidavervirus	unclassified Vidavervirus
Xylella phage Paz	*X. fastidiosa*	*Podoviridae*	43869	KF626666.1	Caudovirales	Autographiviridae	Pradovirus	
Xylella phage Prado	*X. fastidiosa*	*Podoviridae*	43940	KF626667.1	Caudovirales	Autographiviridae	Pradovirus	
Xylella Phage Salvo	*X. fastidiosa*	*Siphoviridae*	55601	KF626668.1	Caudovirales	Siphoviridae	Sanovirus	
Xylella Phage Sano	*X. fastidiosa*	*Siphoviridae*	56147	KF626665.1	Caudovirales	Siphoviridae	Sanovirus	
Xylella phage Xfas53	*X. fastidiosa*	*Podoviridae*	36674	GQ421471.1	Caudovirales	Podoviridae	unclassified Podoviridae	

Table S2. Comparison matrix for similarities between genomes of the phages sequenced in this study. All sequences were assessed by comparing the percentage of shared 22-mers.

1.	2. F C03	3. F C08	4. F C12	5. F C15	6. F C17	7. F C23	8. F C25	9. F C28	10. F C30	11. F C39	12. F C41	13. F C44	14. F C47	15. F C57
16. F C03	17. 1	18. 0.99	19. 0	20. 0.99	21. 0.99	22. 0	23. 0.99	24. 0.25	25. 0.25	26. 0.25	27. 0	28. 0	29. 0.99	30. 0.99
31. F C08	32. 0.99	33. 1	34. 0	35. 0.99	36. 0.99	37. 0	38. 0.99	39. 0.25	40. 0.25	41. 0.25	42. 0	43. 0	44. 0.99	45. 0.99
46. F C12	47. 0	48. 0	49. 1	50. 0	51. 0	52. 0	53. 0	54. 0	55. 0	56. 0	57. 0	58. 0	59. 0	60. 0

61. **F C15**	62. 0.99	63. 0.99	64. 0	65. 1	66. 0.99	67. 0	68. 0.99	69. 0.25	70. 0.25	71. 0.25	72. 0	73. 0	74. 0.99	75. 0.99
76. **F C17**	77. 0.99	78. 0.99	79. 0	80. 1	81. 1	82. 0	83. 1	84. 0.25	85. 0.25	86. 0.25	87. 0	88. 0	89. 1	90. 1
91. **F C23**	92. 0	93. 0	94. 0	95. 0	96. 0	97. 1	98. 0	99. 0	100. 0	101. 0	102. 0	103. 0	104. 0	105. 0
106. **F C25**	107. 0.99	108. 0.99	109. 0	110. 1	111. 1	112. 0	113. 1	114. 0.25	115. 0.25	116. 0.25	117. 0	118. 0	119. 1	120. 1
121. **F C28**	122. 0.25	123. 0.25	124. 0	125. 0.25	126. 0.25	127. 0	128. 0.25	129. 1	130. 0.95	131. 0.75	132. 0	133. 0	134. 0.25	135. 0.25
136. **F C30**	137. 0.25	138. 0.25	139. 0	140. 0.26	141. 0.26	142. 0	143. 0.26	144. 0.95	145. 1	146. 0.75	147. 0	148. 0	149. 0.26	150. 0.26
151. **F C39**	152. 0.25	153. 0.25	154. 0	155. 0.25	156. 0.25	157. 0	158. 0.25	159. 0.75	160. 0.75	161. 1	162. 0	163. 0	164. 0.25	165. 0.25
166. **F C41**	167. 0	168. 0	169. 0	170. 0	171. 0	172. 0	173. 0	174. 0	175. 0	176. 0	177. 1	178. 0	179. 0	180. 0
181. **F C44**	182. 0	183. 0	184. 0	185. 0	186. 0	187. 0	188. 0	189. 0	190. 0	191. 0	192. 0	193. 1	194. 0	195. 0
196. **F C47**	197. 0.99	198. 0.99	199. 0	200. 0.99	201. 0.99	202. 0	203. 0.99	204. 0.25	205. 0.25	206. 0.25	207. 0	208. 0	209. 1	210. 0.99
211. **F C57**	212. 0.99	213. 0.99	214. 0	215. 0.99	216. 0.99	217. 0	218. 0.99	219. 0.25	220. 0.25	221. 0.25	222. 0	223. 0	224. 0.99	225. 1

Table S3. Genomic information used for MCP and Terminase_lsu phylogeny (Fig. S3).

Phage name	Host	Family	Size (nt)	GenBank	Major Capsid Protein ID	Terminase Large Subunit Protein ID
Stenotrophomonas phage DLP4	*S. maltophilia* D1585	*Siphoviridae*	63945	MG018224.1	ATS92208.1	ATS92199.1
Stenotrophomonas phage IME13	*S. maltophilia*	*Myoviridae*	162327	JX306041.1	AFQ22650.1	AFQ22643.1
Stenotrophomonas phage IME15	*S. maltophilia*	*Podoviridae*	38513	JX872508.1	AFV51470.1	AFV51483.1
Stenotrophomonas phage IME-SM1	*S. maltophilia*	*unclassified*	159514	KR560069.1	AKO61665.1	AKO61658.1
Stenotrophomonas phage S1	*S. maltophilia*	*Siphoviridae*	40287	EU849489.1	ACJ24732.1	ACJ24728.1
Stenotrophomonas phage Smp131	*S. maltophilia*	*Myoviridae*	33525	JQ809663.1	AFJ75479.1	AFJ75477.1
Stenotrophomonas phage vB_SmaS-DLP_1	*S. maltophilia* D1585	*Siphoviridae*	42887	KR537872.1	AKI28799.1	AKI28793.1
Stenotrophomonas phage vB_SmaS-DLP_2	*S. maltophilia* D1585	*Siphoviridae*	42593	KR537871.1	AKI28741.1	AKI28735.1
Stenotrophomonas phage vB_SmaS-DLP_6	*S. maltophilia* D1571	*Myoviridae*[1]	168489	KU682439.2	AMQ65921.1	AMQ65913.1
Xanthomonas phage Carpasina	*X. campestris*	*Myoviridae*	61939	MH059633.1	AWD92405.1	AWD92396.1
Xanthomonas phage CP1	*X. axonopodis* pv. *citri*	*Siphoviridae*	43870	AB720063.2	BAM29080.1	BAM29076.1
Xanthomonas phage CP2	*X. axonopodis* pv. *citri*	*Podoviridae*	42963	AB720064.1	BAM66438.1	BAM66434.1
Xanthomonas phage f20-Xaj	*X. arboricola* pv. *juglandis* X-J303	*Podoviridae*	43851	NC_030928.1	YP_009275467.1	YP_009275454.1

Xanthomonas phage f29-Xaj	*X. arboricola* pv. *juglandis* X-J303	*Siphoviridae*	41865	KU595434.1	AMM44781.1	AMM44766.1
Xanthomonas phage f30-Xaj	*X. arboricola* pv. *juglandis* X-J303	*Podoviridae*	44262	NC_030937.1	YP_009276308.1	YP_009276349.1
Xanthomonas phage KPhi1	*X. euvesicatoria*	*Myoviridae*	46077	KY210139.1	APQ41901.1	APQ41906.1
Xanthomonas phage OP1	*X. oryzae* pv. *oryzae*	*Siphoviridae*	43785	AP008979.1	BAE72712.1	BAE72709.1
Xanthomonas phage OP2	*X. oryzae* pv. *oryzae*	*Myoviridae*	46643	AP008986.1	BAE72772.1	BAE72777.1
Xanthomonas phage phi Xc10	*X. campestris* pv. *citri*	*Podoviridae*	44597	MF375456.1	ASZ72035.1	ASZ72044.1
Xanthomonas phage phiL7	*X. campestris* pv. *campestris*	*Siphoviridae*	44080	EU717894.1	ACE75746.1	ACE75743.1
Xanthomonas phage vB_XveM_DIBBI	*X. vesicatoria*	*Myoviridae*	49981	JN022534.1	AEX65675.1	AEX65672.1
Xanthomonas phage XacN1	*X. citri*	*Myoviridae*	384670	AP018399.1	BBA65422.1	BBA65403.1
Xanthomonas phage XAJ2	*X. arboricola* pv. *juglandis*	*Siphoviridae*	49241	KU197014.1	AMW36127.1	AMW36122.1
Xanthomonas phage XAJ24	*X. arboricola* pv. *juglandis*	*Podoviridae*	44861	KU197013.1	AMW36095.1	AMW36109.1
Xanthomonas phage Xoo-sp2	*X. oryzae* pv. *oryzae*	*Siphoviridae*	60497	KX241618.1	ANT45254.1	ANT45236.1
Xanthomonas phage Xop411	*X. oryzae*	*Siphoviridae*	44520	DQ777876.1	ABK00157.1	ABK00154.1
Xanthomonas phage Xp10	*X. oryzae*	*Siphoviridae*	44373	AY299121.1	AAP58676.1	AAP58673.1
Xanthomonas phage Xp15	*X. campestris* pv. *pelargonii*	*Siphoviridae*	55770	AY986977.1	AAX84853.1	AAX84927.1
Xanthomonas phage XPP1	*X. oryzae* pv. *oryzae*	*Myoviridae*	46195	MG944227.1	AVO23667.1	AVO23662.1
Xanthomonas phage XPP2	*X. oryzae* pv. *oryzae*	*Myoviridae*	46480	MG944228.1	AVO23722.1	AVO23717.1
Xanthomonas phage XPP3	*X. oryzae* pv. *oryzae*	*Myoviridae*	49612	MG944229.1	AVO23799.1	AVO23794.1
Xanthomonas phage XPP4	*X. oryzae* pv. *oryzae*	*Myoviridae*	47397	MG944230.1	AVO23889.1	AVO23884.1
Xanthomonas phage XPP6	*X. oryzae* pv. *oryzae*	*Myoviridae*	46281	MG944231.1	AVO23998.1	AVO23993.1
Xanthomonas phage XPP8	*X. oryzae* pv. *oryzae*	*Myoviridae*	46278	MG944232.1	AVO24023.1	AVO24028.1
Xanthomonas phage XPP9	*X. oryzae* pv. *oryzae*	*Myoviridae*	48669	MG944233.1	AVO24107.1	AVO24102.1
Xanthomonas phage XPV1	*X. oryzae* pv. *oryzae*	*Myoviridae*	46503	MG944234.1	AVO24222.1	AVO24217.1
Xanthomonas phage XPV2	*X. oryzae* pv. *oryzae*	*Myoviridae*	45969	MG944235.1	AVO24254.1	AVO24259.1
Xanthomonas phage XPV3	*X. oryzae* pv. *oryzae*	*Myoviridae*	47046	MG944236.1	AVO24390.1	AVO24319.1
Xylella phage Paz	*X. fastidiosa*	*Podoviridae*	43869	KF626666.1	AHB12129.1	AHB12142.1
Xylella phage Prado	*X. fastidiosa*	*Podoviridae*	43940	KF626667.1	AHB12182.1	AHB12195.1
Xylella Phage Salvo	*X. fastidiosa*	*Siphoviridae*	55601	KF626668.1	AHB12256.1	AHB12262.1
Xylella Phage Sano	*X. fastidiosa*	*Siphoviridae*	56147	KF626665.1	AHB12081.1	AHB12087.1
Xylella phage Xfas53	*X. fastidiosa*	*Podoviridae*	36674	GQ421471.1	ACV41134.1	ACV41131.1

For phylogenetic analysis, protein sequences of the Major Capsid Protein (MCP) and Terminase Large subunit (Terminase_lsu) of the *Caudovirales* bacteriophages infecting *Xanthomonadales* were downloaded from the Genbank database (Table S2). Sequence alignments were performed using ClustalW, and cladograms were built using the MEGA-X software [1]. Aminoacid sequence data set were concatenated using Mesquite Version 2.6 [2]. Then, trees were inferred based on the maximum-likelihood Statistical method using MEGA-X and bootstrap values were calculated from 1,000 bootstrap replications. Genetic distances were determined by the Jones-Taylor-Thornton (JTT) model [3].

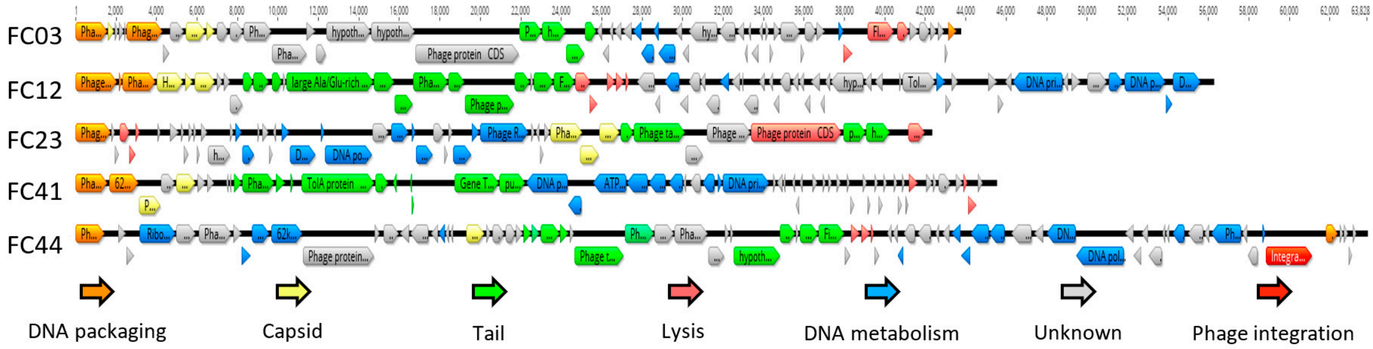

Figure S1. Genomic organization and main functions encoded by FC03-Usme, FC12-Bacata, FC23-Cota, FC41-Suba and FC44-Bosa phage genomes, the type phages of the 5 PCR groups defined in this study. .

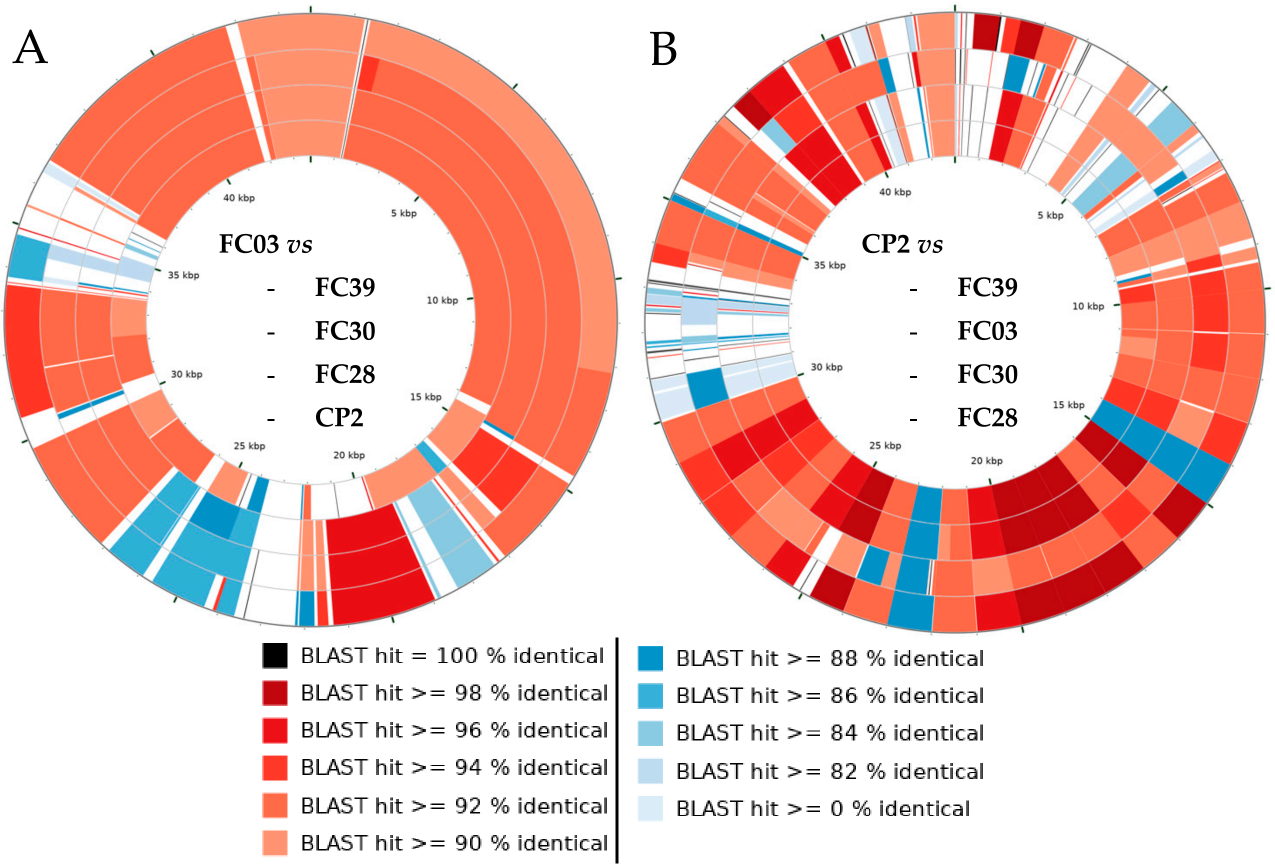

Figure S2. Visualization of BLAST-pairwise comparisons of genome sequences of FCO3-Usme-like phages and *Xanthomonas axonopodis* phage CP2. Genomic sequences of isolated phage FC03-Usme, and *X. axonopodis* phage CP2 were cut in 1 kb-length fragments. Conservation of each fragment of a query genome was searched in the others using the BLASTN algorithm. The CGVIEW analytic tool [4] was used to represent the results, with the order of phage genome given from the external to the internal circles. The color scale indicates the level of identity. White stripes indicate fragments from the query genome that are absent in the others. A- Pairwise comparisons of FC03-Usme versus FC39-Tenjo, FC30-Tabio, FC28-Sopo and *X. axonopodis* phage CP2; B- Pairwise comparisons of *X. axonopodis* phage CP2 versus FC39-Tenjo, FC03-Usme, FC30-Tabio and FC28-Sopo.

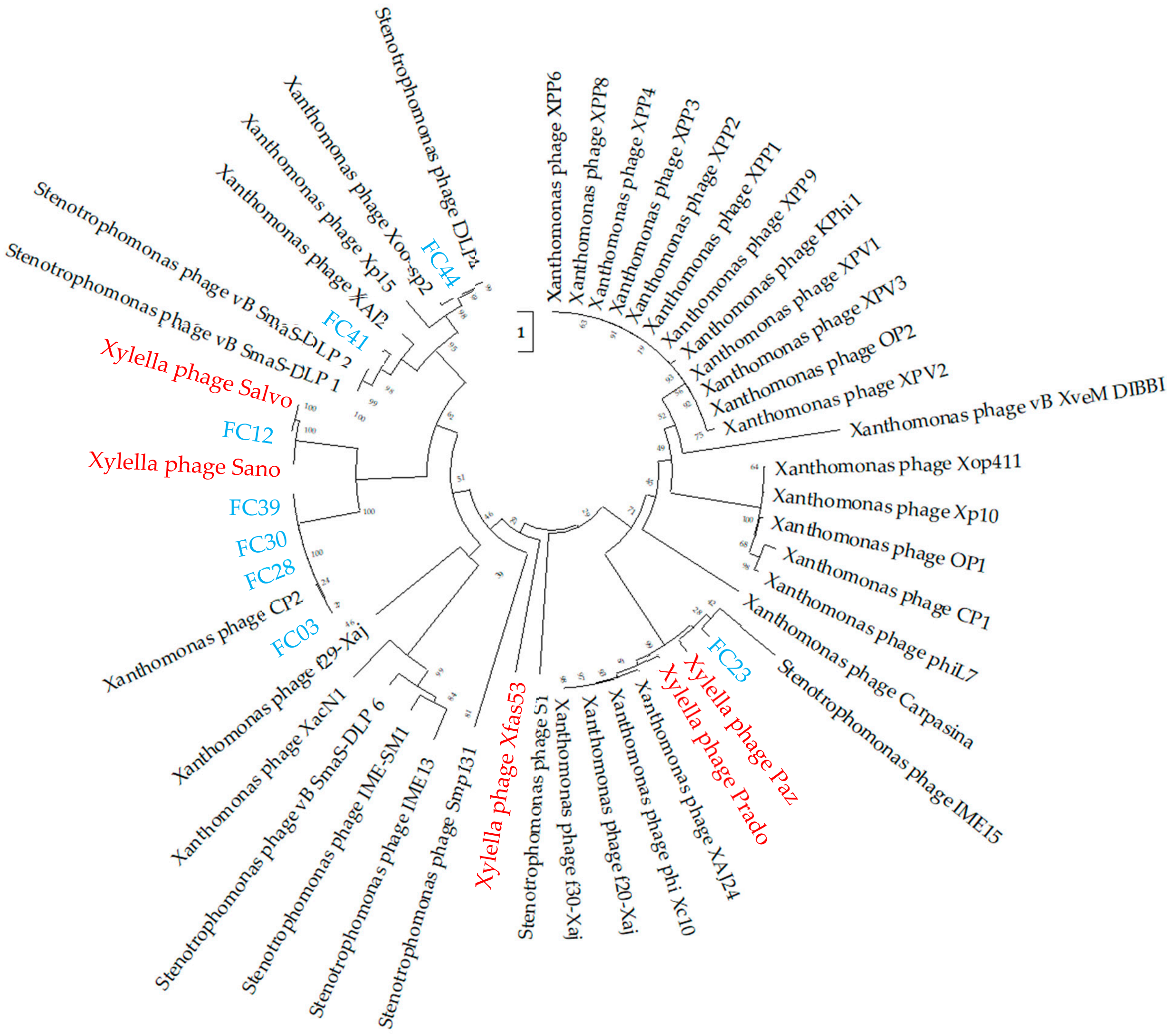

Figure S3. Phylogeny of phages infecting Xanthomonads. Circular cladograms showing the relationships between amino acid sequences obtained in this study and the isolated Caudovirales phages active on various species of the genera Xanthomonas and Stenotrophomonas. The Neighbor Joining trees based on the concatenated alignment of the conserved Major Capsid Protein and the Terminase large subunit protein with the highest log likelihood - (-100895.80) are shown. Initial tree for the heuristic search was obtained automatically by applying Neighbor-Join to a matrix of pairwise distances estimated using the JTT model, and then selecting the topology with superior log likelihood value. This analysis involved 51 amino acid sequences. A total of 2214 positions were analyzed in the final dataset. Numbers indicated on branches indicate the number of substitutions per site. Red labels highlight referenced bacteriophages that are active on X. fastidiosa and blue labels highlight bacteriophages isolated in this study.

Supplemental References:

1. Kumar, S.; Stecher, G.; Li, M.; Knyaz, C.; Tamura, K. MEGA X: Molecular Evolutionary Genetics Analysis across Computing Platforms. *Mol Biol Evol* **2018**, *35*, 1547–1549, doi:10.1093/molbev/msy096.
2. Maddison, W.; Maddison, D. Mesquite: A Modular System for Evolutionary Analysis. Version 2.6 Available online: /paper/Mesquite%3A-a-modular-system-for-evolutionary-Version-Maddison-Maddison/7a6142cfa79cc01ceced5e144bd0e01a0f241a74 (accessed on 20 September 2020).
3. Jones, D.T.; Taylor, W.R.; Thornton, J.M. The Rapid Generation of Mutation Data Matrices from Protein Sequences. *Bioinformatics* **1992**, *8*, 275–282, doi:10.1093/bioinformatics/8.3.275.
4. Stothard, P.; Wishart, D.S. Circular Genome Visualization and Exploration Using CGView. *Bioinformatics* **2005**, *21*, 537–539, doi:10.1093/bioinformatics/bti054.
